# Correction: Biomarkers of professional cybersportsmen: Event related potentials and cognitive tests study

**DOI:** 10.1371/journal.pone.0335226

**Published:** 2025-10-22

**Authors:** Sergei Gostilovich, Airat Kotliar Shapirov, Andrei Znobishchev, Anh-Huy Phan, Andrzej Cichocki

There are errors in the Funding section. The correct Funding statement is: This study was partially funded by the Russian Federation’s Ministry of Education and Science (grant 075.10.2021.068).
