# Supplementary material for: Biomarkers of professional cybersportsmen: Event related potentials and cognitive tests study
Source: PLoS One. 2023 Aug 1;18(8):e0289293. doi: 10.1371/journal.pone.0289293 (PMC10393144; doi:10.1371/journal.pone.0289293)
Supplement: S1 Appendix — (PDF) [file pone.0289293.s001.pdf]

S1 Appendix. Summary table of EEG studies

Table S1.1. EEG studies literature summary

Link	Compared Groups (number of members of the groups)	Experiment stimuli	Results overview	Claimed conclusion
Hamon at al. [8]	Control group vs witnesses vs students in physical and athletic education vs sprinters (8 in each group)	Oddball auditory stimuli	ERP: Greater N100, P200, and P3 amplitude for athletes (physical and athletic education students and sprinters)	The differences indicate a greater adaptative reactivity of attentional mechanisms in athletes.
Hung at al. [10]	Table tennis players vs control group (15 vs 15)	Posner's cued attention task	ERP: Table tennis players had an inverse cueing effect for N100 amplitude (i.e., amplitude of N100 to the invalid cue > amplitude of N100 to the valid cue), while the control group did not.	Table tennis players' superior reactivity to stimuli occurring in uncertain positions.
Taliep at al. [11]	Skilled and less-skilled cricket batsmen (8 vs 10)	Videos of action of cricket game	ERP: P300 latency and amplitude were significantly different for the two groups and can be separated by Logistic regression	Superior perceptual decision-making ability of skilled sportsmen.
Sanchez-Lopez at al. [12]	Skilled and novice martial arts athletes (11 vs 10)	Continuous performance task	ERP: Skilled athletes showed significant amplitude differences between target and non-target conditions in P100 and P200	Better-controlled attention in skilled athletes.
Jin at al. [13]	Badminton players and control group (18 Vs 18)	Videos of badminton games	ERP: Badminton players showed enlarged amplitudes of P300 and P200	Superior action anticipation intrinsic to professional badminton players.
Percio at al. [14]	Elite karate athletes vs amateur karate athletes vs non-athletes (17 vs 14 vs 15)	Pictures with basket and karate attacks	ERP: Karate athletes (elite and amateur) had a lower amplitude of P300 and P400 between the basket and karate attacks.	Elite athletes have improved sustained visuo-spatial attention and self-control.
Taddei at al. [44]	Young fences vs middle-age fences vs young non-athletes vs middle-age athletes (10 in each group)	Visual motor tasks	ERP: N200 component of fencers had shorter latencies and larger amplitudes than non-athletes, N100 and P300 components were enhanced in fencers independent of age.	The practice of open-skill sports is associated with improvement of the executive functions.
Kao at al. [20]	Golfers players (18)	Performing 100 putts	EEG spectral: Midline theta power was lower for best putts	Optimal attentional engagement, as characterized by a lower midline theta power, is beneficial for successful skilled performance.
Loze at al. [21]	Professional air-pistol shooters (6)	Performing 60 shots	EEG spectral: alpha power increased before the best shots and decreased before the worst shots	Visual attention to the pistol and target was suppressed during the pre-shot period of best shots.
Irak et al. [38]	Excessive video game players vs non-players (18 vs 18)	N-back task	ERP: P100 and P200 amplitudes were higher for the video game players, P300 amplitude were not statistically different	Excessive video game playing does not cause a deterioration in this type of attention and memory performance.

Sepúlveda at al. [39]	Experienced video game players vs no video game players (12 vs 12)	Videos of FPS gameplay	EEG spectral: the experienced players showed an increased right intrahemispheric prefrontal-parietal correlation (F4-P4 electrodes) in the gamma band	The detected superior coupling between the prefrontal and parietal cortices could represent a characteristic pattern of brain functionality in experienced players as they make motor representations.
Ding at al. [40]	Professional vs semi-professional vs novice players in MOBA game League of Legends (10 vs 10 vs 20)	Playing the game<br><br>(Also there were cognitive tests).	EEG spectral: Using the neural features derived from EEG theta and alpha power, all three groups can be well separated with higher classification accuracies (>88.24%)	It is possible to recognize MOBA expertise using neural measurements.

List of abbreviations:
EEG - electroencephalogram
ERP - event-related potential
FPS - first-person shooter
MOBA - multiplayer online battle arena
