# Supplementary material for: Biomarkers of professional cybersportsmen: Event related potentials and cognitive tests study
Source: PLoS One. 2023 Aug 1;18(8):e0289293. doi: 10.1371/journal.pone.0289293 (PMC10393144; doi:10.1371/journal.pone.0289293)
Supplement: S3 Appendix — (PDF) [file pone.0289293.s003.pdf]

S3 Appendix. Cognitive tests description

RTM and RTK tests

A red circle will show up after a random time (e.g., from 2 to 5 seconds) at the center of the screen. After the participant sees the circle, he/she should click the mouse (for RTM) or press the space button (for RTK) as soon as possible. The participant interacts with the mouse with the right hand and the keyboard with the left hand as if he/she is playing in CS:GO. As an example, the view of the RTM test is presented in Figure S3.1.

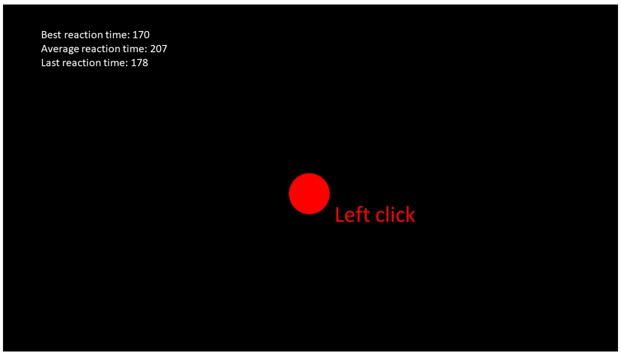

Figure S3.1: View of RTM test.

RTD test

A red or a blue circle (each with a 50% chance) will show up after a random time (e.g., from 2 to 5 seconds) at the center of the screen. After the participant sees the circle, he/she should, as soon as possible, click the appropriate mouse button: left click for red circles and right click for blue circles. The participant interacts with the mouse with the right hand as if he/she is playing in CS:GO. As an example, the view of the RTD test is presented in Figure S3.2.

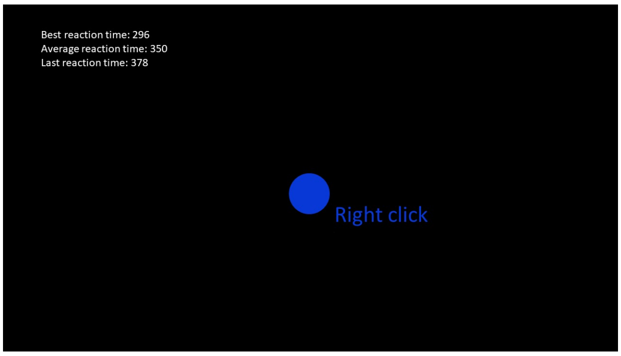

Figure S3.2: View of RTD test during presenting of a blue circle.

VS test

A matrix of letters is shown to the participant at the center of the screen. 50% of which are filled with letters "T", and the remaining 50% are empty. Additionally, one letter "L" can be presented in the matrix with a 50% chance. The subject has to click the left mouse button if he/she believes that the letter "L" is presented or the right mouse button otherwise. In the conducted experiments, the size of the matrix was 19x19. The view of VS test is presented in Figure S3.3

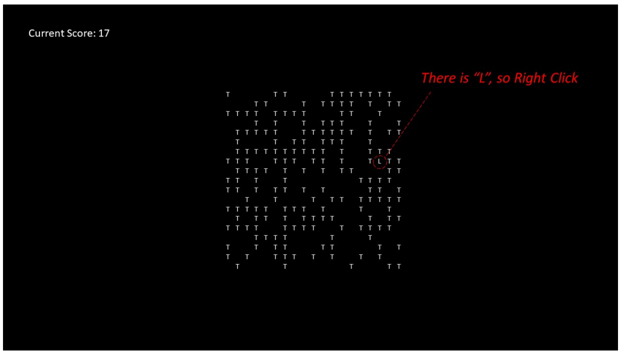

Figure S3.3: View of VS test.
