# Supplementary material for: Biomarkers of professional cybersportsmen: Event related potentials and cognitive tests study
Source: PLoS One. 2023 Aug 1;18(8):e0289293. doi: 10.1371/journal.pone.0289293 (PMC10393144; doi:10.1371/journal.pone.0289293)
Supplement: S4 Appendix — (PDF) [file pone.0289293.s004.pdf]

S4 Appendix. Topographical spectrums

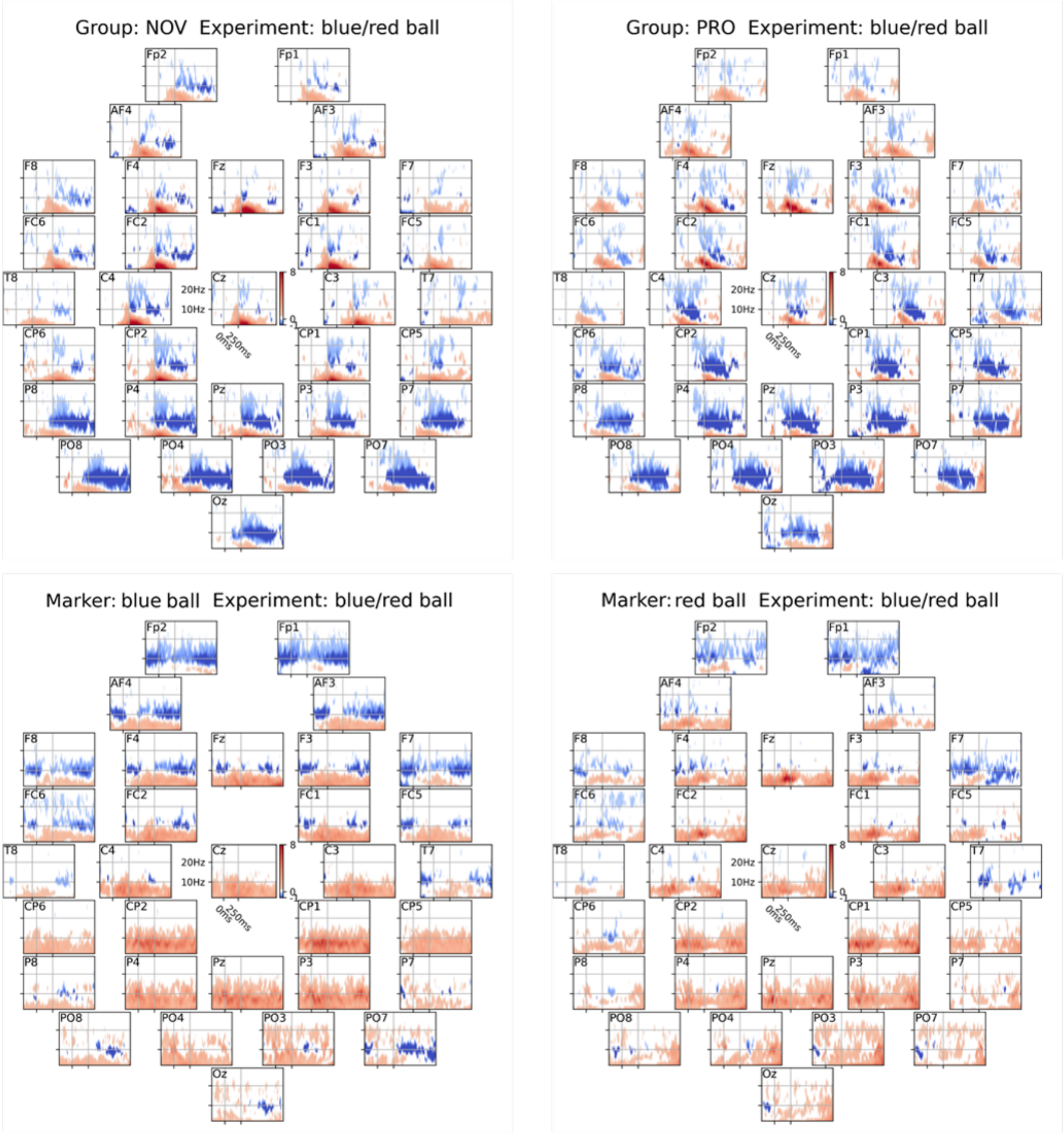

Figure S4.1: Topographical spectrums for Blue/Red Ball paradigm. Top row compares ERP spectrums (target vs non-target) for novices (NOV, left) and professionals (PRO, right). Bottom row compares spectrums of these groups (PRO vs NOV) for blue ball (non-target, left) and red ball (target, right) markers. Statistically non-significant regions (p>0.05 for nonparametric TFCE) are colored white. Significant regions (p<0.05) are colored as difference in mean spectrograms for target vs. non-target (top) and PRO vs. NOV (bottom).

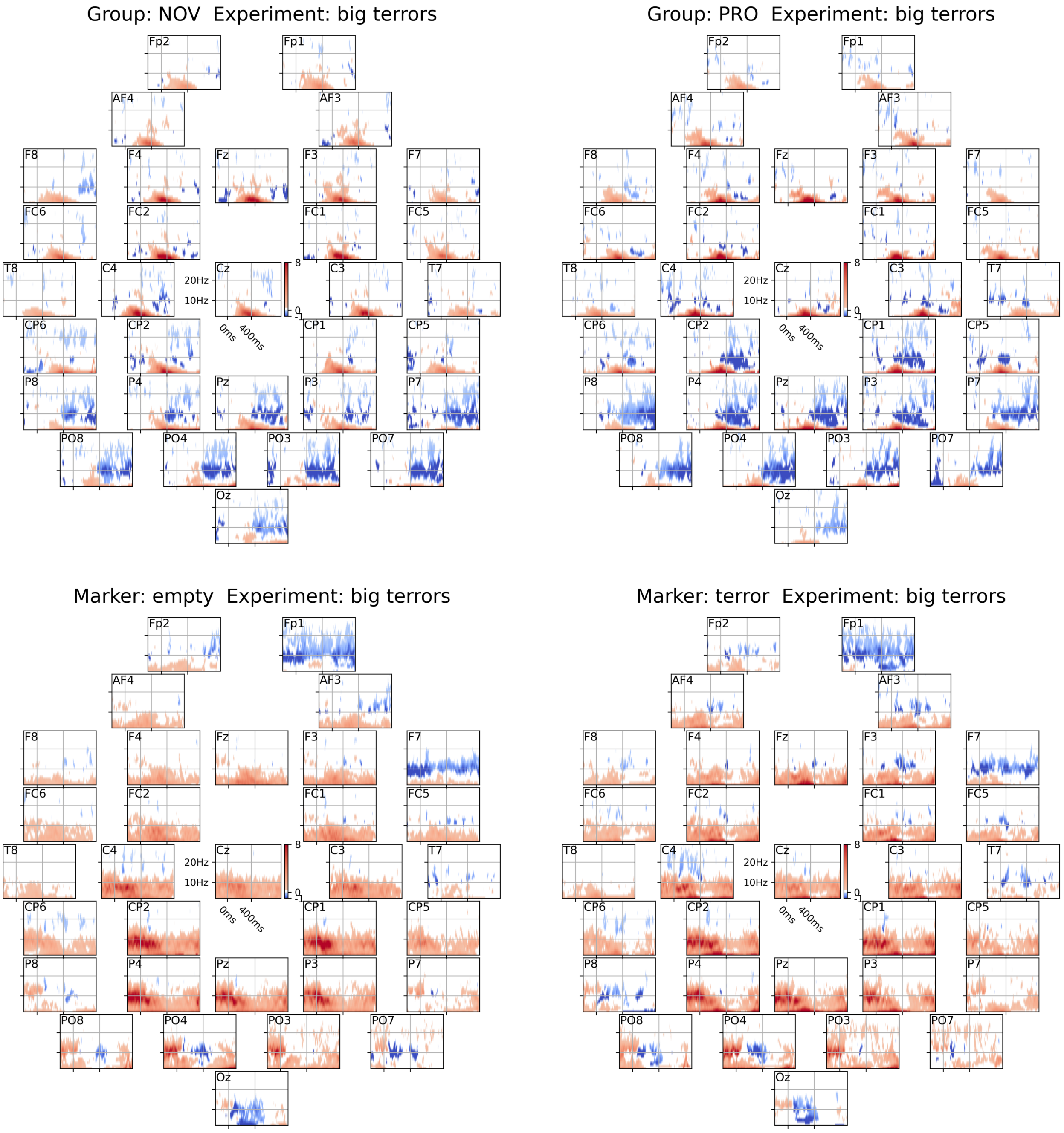

Figure S4.2: Topographical spectrums for Big Terrors paradigm. Top row compares ERP spectrums (target vs non-target) for novices (NOV, left) and professionals (PRO, right). Bottom row compares spectrums of these groups (PRO vs NOV) for empty (non-target, left) and terror (target, right) markers. Statistically non-significant regions ($p>0.05$ for nonparametric TFCE) are colored white. Significant regions ($p<0.05$) are colored as difference in mean spectrograms for target vs. non-target (top) and PRO vs. NOV (bottom).

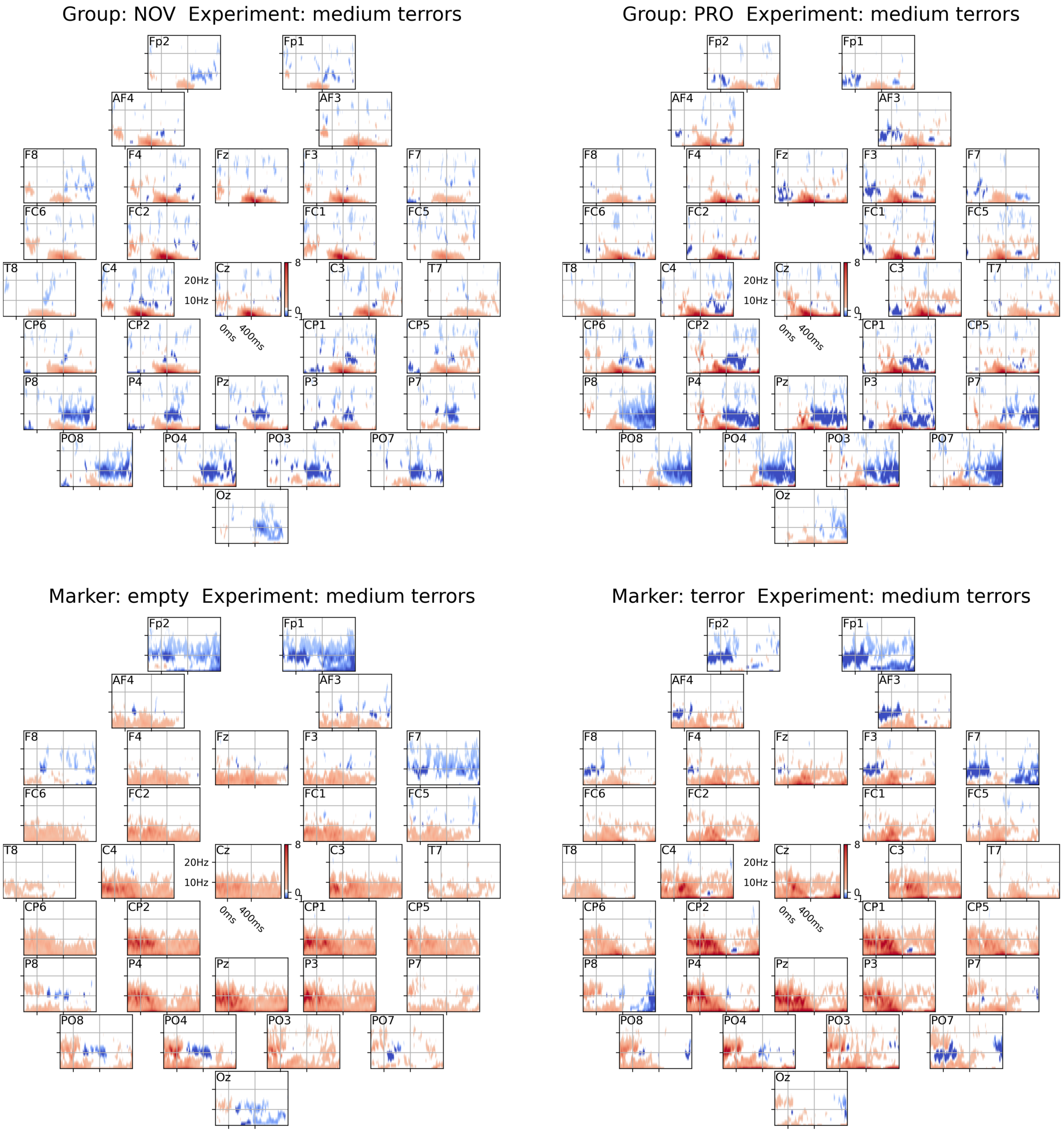

Figure S4.3: Topographical spectrums for Medium Terrors paradigm. Top row compares ERP spectrums (target vs non-target) for novices (NOV, left) and professionals (PRO, right). Bottom row compares spectrums of these groups (PRO vs NOV) for empty (non-target, left) and terror (target, right) markers. Statistically non-significant regions (p>0.05 for nonparametric TFCE) are colored white. Significant regions (p<0.05) are colored as difference in mean spectrograms for target vs. non-target (top) and PRO vs. NOV (bottom).

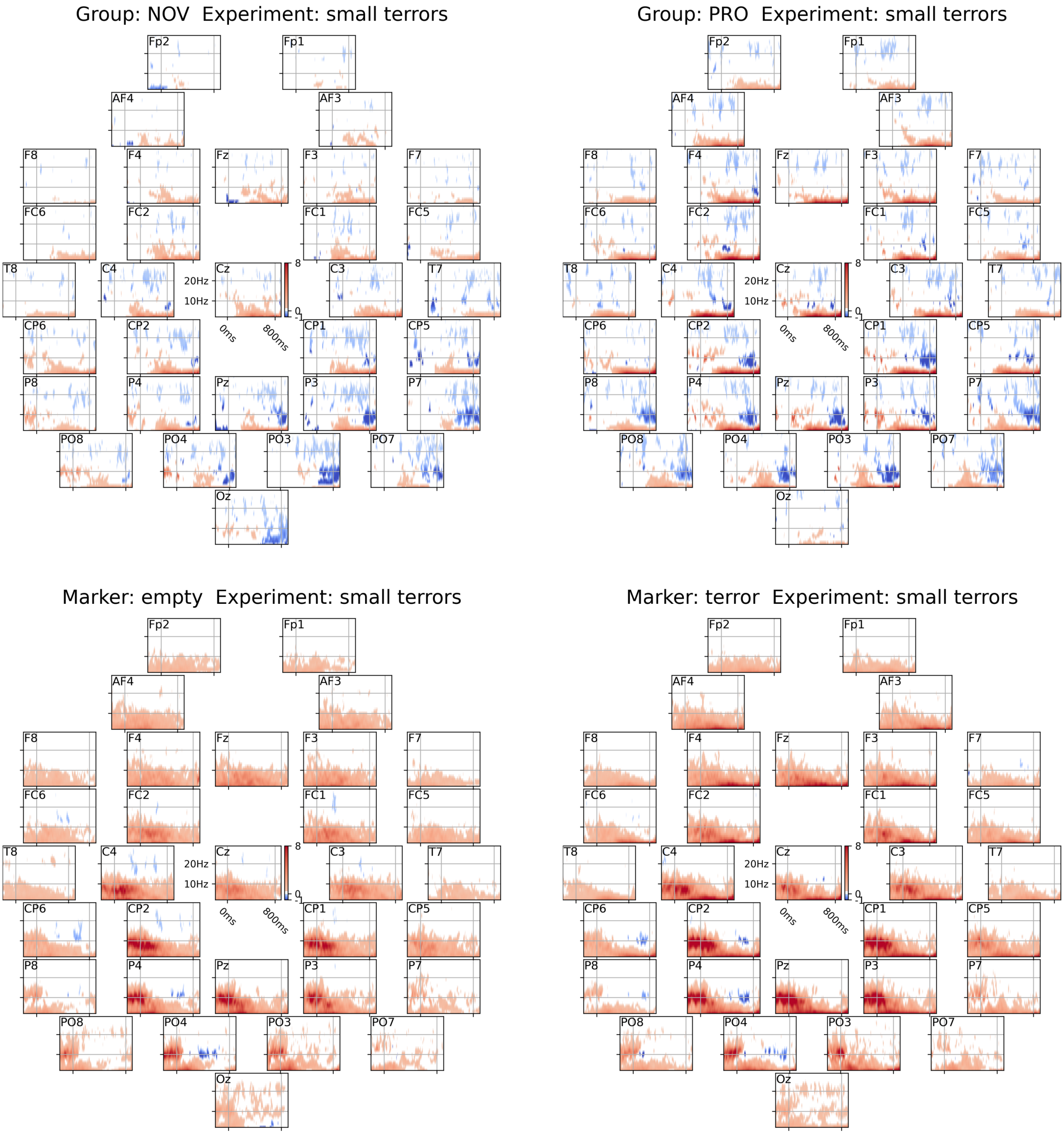

Figure S4.4: Topographical spectrums for Small Terrors paradigm. Top row compares ERP spectrums (target vs non-target) for novices (NOV, left) and professionals (PRO, right). Bottom row compares spectrums of these groups (PRO vs NOV) for empty (non-target, left) and terror (target, right) markers. Statistically non-significant regions ($p>0.05$ for nonparametric TFCE) are colored white. Significant regions ($p<0.05$) are colored as difference in mean spectrograms for target vs. non-target (top) and PRO vs. NOV (bottom).
