# Supplementary material for: Biomarkers of professional cybersportsmen: Event related potentials and cognitive tests study
Source: PLoS One. 2023 Aug 1;18(8):e0289293. doi: 10.1371/journal.pone.0289293 (PMC10393144; doi:10.1371/journal.pone.0289293)
Supplement: S5 Appendix — (PDF) [file pone.0289293.s005.pdf]

S5 Appendix. ERP components peak amplitude and latency distribution

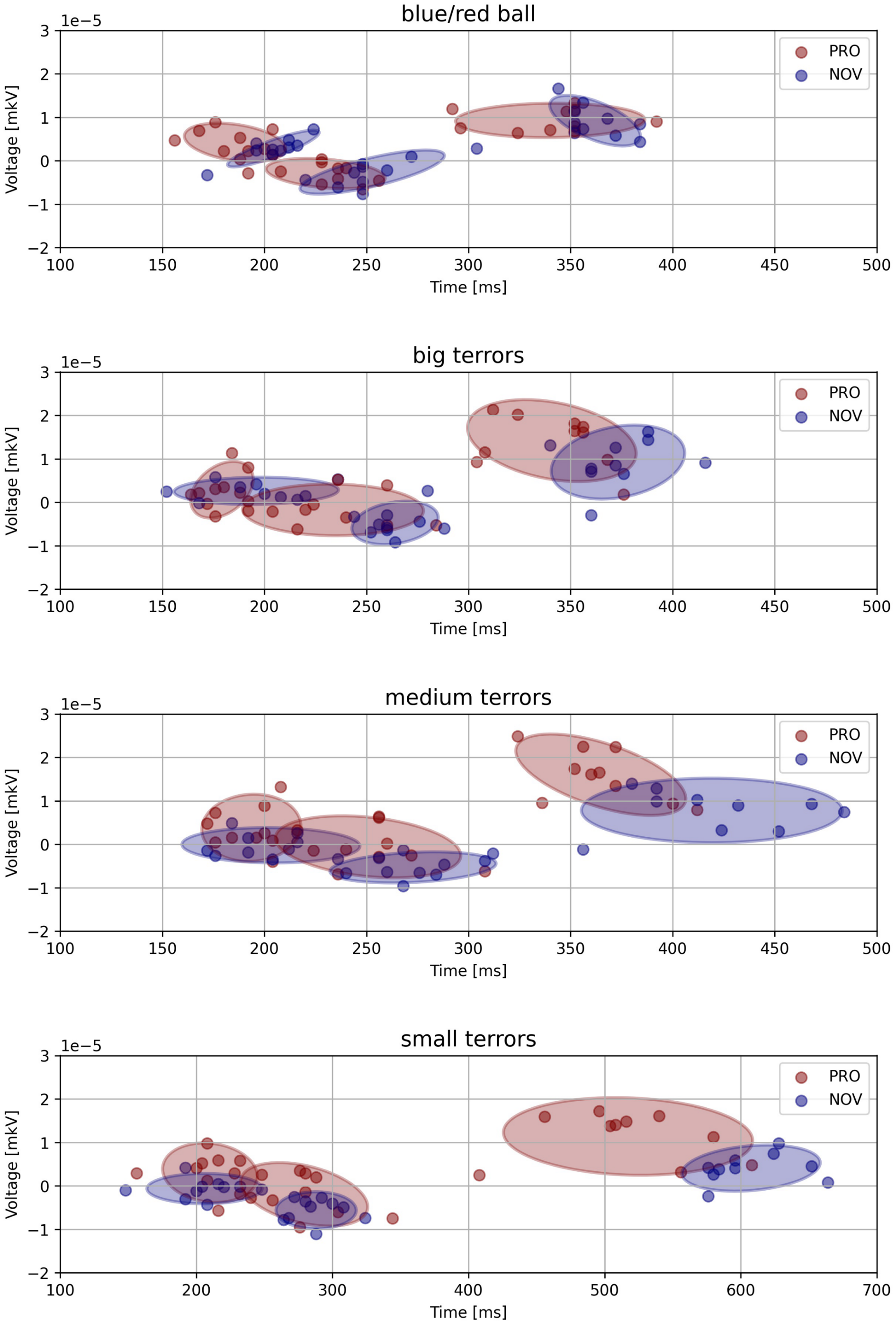

Figure S5.1: Latency and amplitude distribution of peaks of ERP components for professionals (PRO) and novices (NOV).
