# Supplementary material for: Biomarkers of professional cybersportsmen: Event related potentials and cognitive tests study
Source: PLoS One. 2023 Aug 1;18(8):e0289293. doi: 10.1371/journal.pone.0289293 (PMC10393144; doi:10.1371/journal.pone.0289293)
Supplement: S6 Appendix — (PDF) [file pone.0289293.s006.pdf]

S6 Appendix. Correlation analysis

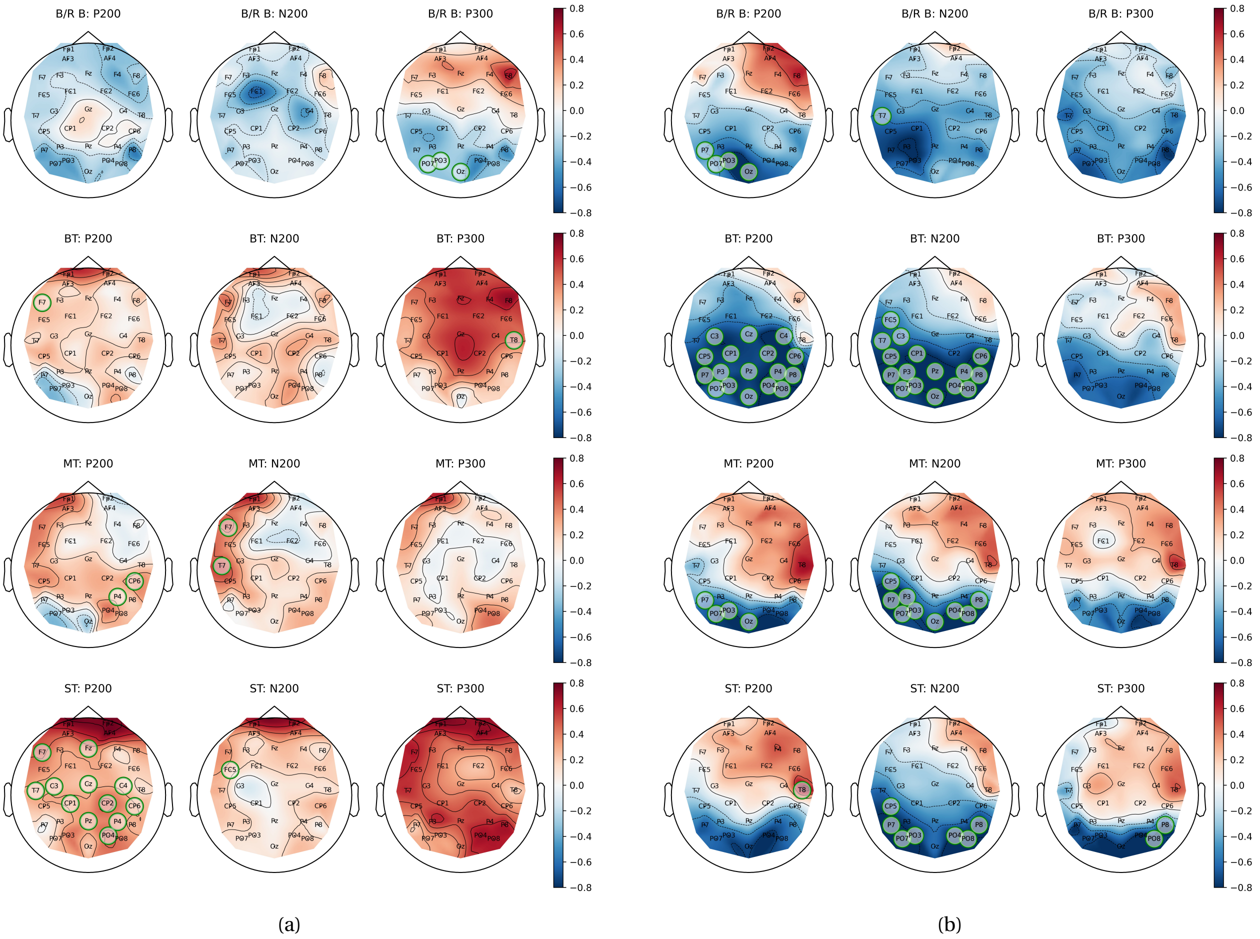

Figure S6.1: Topomaps of correlations between correct rate in RTD and mean amplitudes of ERP components for PRO (a) and NOVICE (b) groups. Columns represent ERP components: P200, N200, and P300. Rows represent EEG paradigms: Blue/Red Ball (B/RB), Big Terrors (BT), Medium Terrors (MT), and Small Terrors (ST). The statistically significant channels are highlighted by green circles.

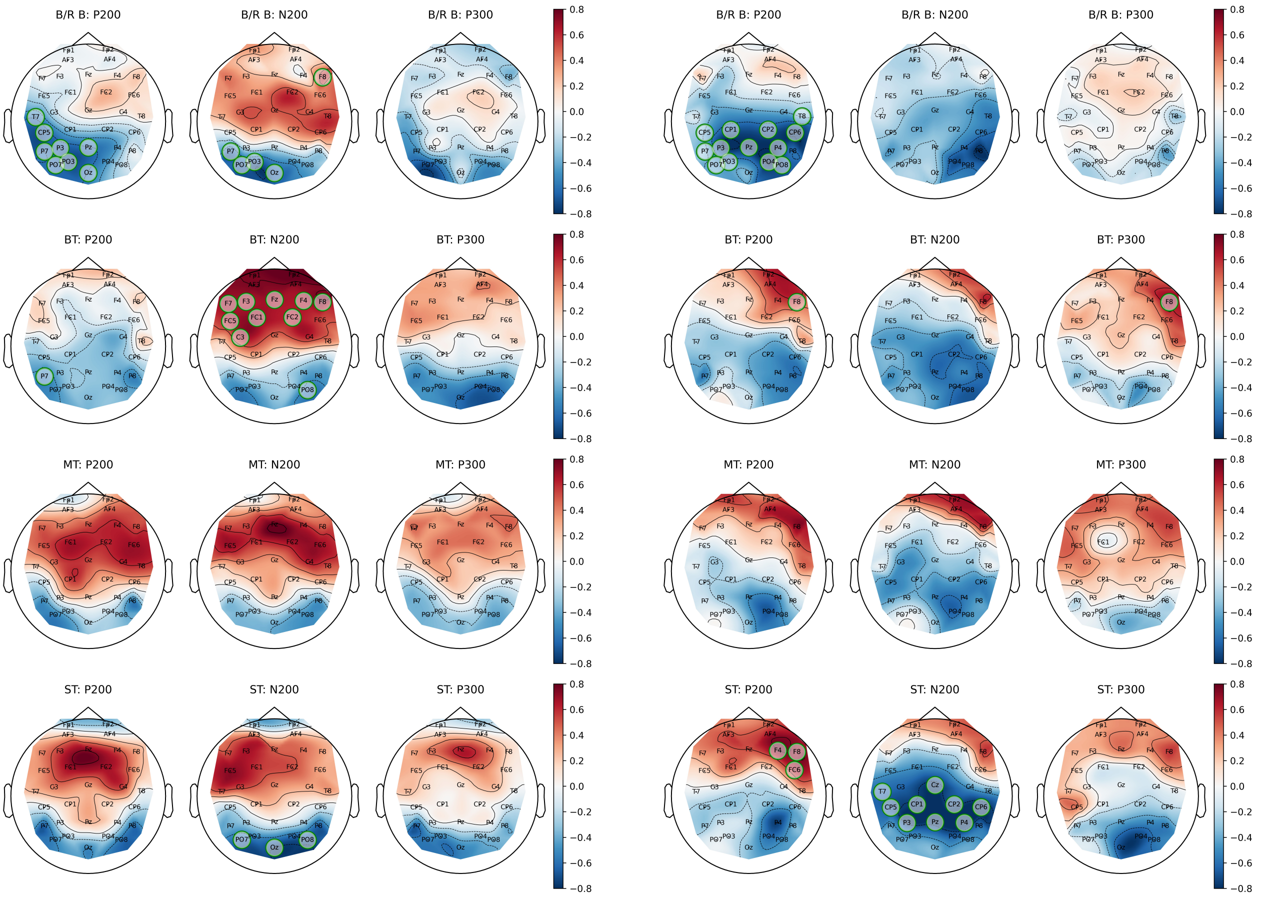

(a) (b)

Figure S6.2: Topomaps of correlations between final score in VS and mean amplitudes of ERP components for PRO (a) and NOVICE (b) groups. Columns represent ERP components: P200, N200, and P300. Rows represent EEG paradigms: Blue/Red Ball (B/RB), Big Terrors (BT), Medium Terrors (MT), and Small Terrors (ST). The statistically significant channels are highlighted by green circles.

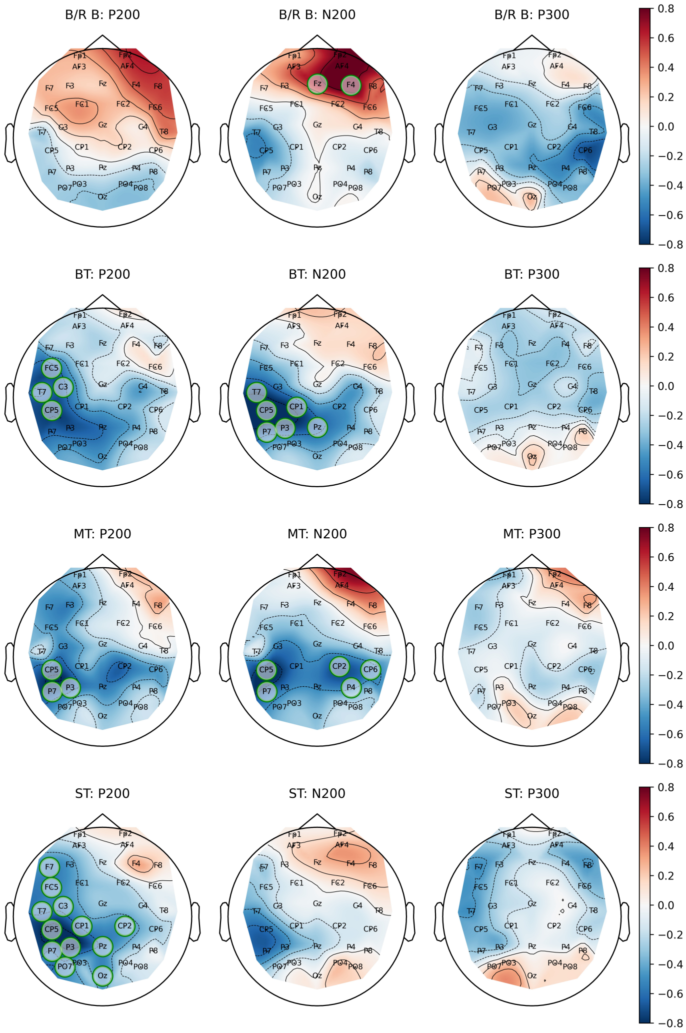

Figure S6.3: Topomaps of correlations between hours spent in CS:GO and mean amplitudes of ERP components for professional players. Columns represent ERP components: P200, N200, and P300. Rows represent EEG paradigms: Blue/Red Ball (B/RB), Big Terrors (BT), Medium Terrors (MT), and Small Terrors (ST). The statistically significant channels are highlighted by green circles.
