# Supplementary material for: Biomarkers of professional cybersportsmen: Event related potentials and cognitive tests study
Source: PLoS One. 2023 Aug 1;18(8):e0289293. doi: 10.1371/journal.pone.0289293 (PMC10393144; doi:10.1371/journal.pone.0289293)
Supplement: S1 Data — (ZIP) [file pone.0289293.s007.zip › S1 Appendix.docx]

**S1 Appendix. Summary table of EEG studies**

**Table S1.1. EEG studies literature summary**

| Link | Compared Groups (number of members of the groups) | Experiment stimuli | Results overview | Claimed conclusion |
| --- | --- | --- | --- | --- |
| Hamon at al. [8] | Control group vs witnesses vs students in physical and athletic education vs sprinters (8 in each group) | Oddball auditory stimuli | ERP: Greater N100, P200, and P3 amplitude for athletes (physical and athletic education students and sprinters) | The differences indicate a greater adaptative reactivity of attentional mechanisms in athletes. |
| Hung at al. [10] | Table tennis players vs control group (15 vs 15) | Posner’s cued attention task | ERP: Table tennis players had an inverse cueing effect for N100 amplitude (i.e., amplitude of N100 to the invalid cue > amplitude of N100 to the valid cue), while the control group did not. | Table tennis players’ superior reactivity to stimuli occurring in uncertain positions. |
| Taliep at al. [11] | Skilled and less-skilled cricket batsmen  (8 vs 10) | Videos of action of cricket game | ERP: P300 latency and amplitude were significantly different for the two groups and can be separated by Logistic regression | Superior perceptual decision-making ability of skilled sportsmen. |
| Sanchez-Lopez at al. [12] | Skilled and novice martial arts athletes (11 vs 10) | Continuous performance task | ERP: Skilled athletes showed significant amplitude differences between target and non-target conditions in P100 and P200 | Better-controlled attention in skilled athletes. |
| Jin at al. [13] | Badminton players and control group (18 Vs 18) | Videos of badminton games | ERP: Badminton players showed enlarged amplitudes of P300 and P200 | Superior action anticipation intrinsic to professional badminton players. |
| Percio at al. [14] | Elite karate athletes vs amateur karate athletes vs non-athletes (17 vs 14 vs 15) | Pictures with basket and karate attacks | ERP: Karate athletes (elite and amateur) had a lower amplitude of P300 and P400 between the basket and karate attacks. | Elite athletes have improved sustained  visuo-spatial attention and self-control. |
| Taddei at al. [44] | Young fences vs middle-age fences vs young non-athletes vs middle-age athletes (10 in each group) | Visual motor tasks | ERP: N200 component of fencers had shorter latencies and larger amplitudes than non-athletes,  N100 and P300 components were enhanced in fencers independent of age. | The practice of open-skill sports is associated with improvement of the executive functions. |
| Kao at al. [20] | Golfers players (18) | Performing 100 putts | EEG spectral: Midline theta power was lower for best putts | Optimal attentional engagement, as characterized by a lower midline theta power, is beneficial for successful skilled performance. |
| Loze at al. [21] | Professional air-pistol shooters (6) | Performing 60 shots | EEG spectral: alpha power increased before the best shots and decreased before the worst shots | Visual attention to the pistol and target was suppressed during the pre-shot period of best shots. |
| Irak et al. [38] | Excessive video game players vs non-players (18 vs 18) | N-back task | ERP: P100 and P200 amplitudes were higher for the video game players, P300 amplitude were not statistically different | Excessive video game playing does not cause a deterioration in this type of attention and memory performance. |
| Sepúlveda at al. [39] | Experienced video game players vs no video game players (12 vs 12) | Videos of FPS gameplay | EEG spectral: the experienced players showed an increased right intrahemispheric prefrontal-parietal correlation (F4-P4 electrodes) in the gamma band | The detected superior coupling between the prefrontal and parietal cortices could represent a characteristic pattern of brain functionality in experienced players as they make motor representations. |
| Ding at al. [40] | Professional vs semi-professional vs novice players in MOBA game League of Legends (10 vs 10 vs 20) | Playing the game  (Also there were cognitive tests). | EEG spectral: Using the neural features derived from EEG theta and alpha power, all three groups can be well separated with higher classification accuracies (>88.24%) | It is possible to recognize MOBA expertise using neural measurements. |

**List of abbreviations:**

EEG - electroencephalogram

ERP - event-related potential

FPS - first-person shooter

MOBA - multiplayer online battle arena
