# Supplementary material for: Biomarkers of professional cybersportsmen: Event related potentials and cognitive tests study
Source: PLoS One. 2023 Aug 1;18(8):e0289293. doi: 10.1371/journal.pone.0289293 (PMC10393144; doi:10.1371/journal.pone.0289293)
Supplement: S1 Data — (ZIP) [file pone.0289293.s007.zip › S2 Appendix.docx]

**S2 Appendix. Summary table of cognitive skills studies**

**Tabel S2.1 Cognitive skills literature summary**

| Link | Compared Groups | Cognitive skills and tests | Results overview | Claimed conclusion |
| --- | --- | --- | --- | --- |
| Volodchenko at al. [25] | Kick-boxers vs striking, combat martial artists vs wrestling athletes (17 vs 35 vs 24) | - Simple motor skills  - stability to the disorienting, reaction (simple, choice, to a moving objects)  - line reproduction test | Kickboxers showed the best simple motor skills, choice reaction time, and a line’s speed. Striking combat martial artists were in second place.  Other parameters were close for all groups. | The research determined differences in the functional status of kick-boxing athletes in comparison with other martial athletes. |
| Akpinar at al. [26] | Football players of 4 different clubs (20 vs 23 vs 21 vs 16) | - Problem-solving skill | Professional footballers were beyond the mid-high level of problem-solving skills.  There were meaningful differences in their sports club and age variables. | Problem-solving skill is weakly dependent on the sportsmen’s marital status, parents’ educational status and occupation, occupation in the game, etc. |
| Eroglu at al. [27] | Professional orienteering athletes (200) | - Problem-solving skill | Professional orienteering athletes were beyond the mid-high level of problem-solving skills. | The difference between the problem-solving skills of the athletes and their age, marital status, sports age, years of practice in orienteering sports, and the status of being a national player is significant (p<0.05). |
| Elferink-Gemser at al. [28] | Elite vs sub-elite table tennis players (13 vs 13) | - Metacognition  - working memory  - inhibitory control  - cognitive flexibility | Their performance was above the norm.  Elite athletes were better in the inhibitory control test then sub-elites | A relation with performance level has been shown, which may be explained by the greater exposure to table tennis for elite compared to sub-elite players. |
| Bickmann at al. [31] | Professional players vs non-professional players vs non-professional traditional athletes (18 vs 21 vs 36)  (Players were from different game genres) | - Visual reaction time  - acoustic reaction time  - choice reaction | No significant difference in the tested reaction times between the groups.  Players from sports simulations had significantly shorter reaction times than players in MOBA games. | Traditional sports and eSports may improve reaction times to a similar amount. Various game genres require different reaction times or may affect related abilities in different ways. |
| Dobrowolski at al. [32] | FPS players vs RTS players vs non-players (29 vs 30 vs 28) | - Task switching  - multiple-object tracking | Both FPS and RTS players showed better task switching performance than non-players. For RTS players were superior to both other considered groups in multi-object tracking | Video gameplay benefits are related to actions performed during the game |
| Colzato at al. (a) [33] | FPS players vs non-players (17 vs 17) | - Task switching paradigm | The players had smaller switching costs (i.e., greater cognitive flexibility) than non-players. | The work supports the idea that playing FPS games promotes cognitive flexibility |
| Colzato at al. (b) [34] | FPS players vs non-players (26 vs 26) | - N-back task  - stop-signal paradigm | The players were faster in reacting to go signals but have comparable stopping performance. The players also had superior working memory | FPS games are associated with enhanced flexible updating of task-relevant information without affecting impulsivity. |
| Ding at al. [40] | Professional vs semi-professional vs novice players in MOBA game League of Legends (10 vs 10 vs 20) | - Reaction time,  - visual search  -multi-object tracking  (Also there were EEG experiments) | The significant difference between the groups was only for the multi-object tracking test.  Professional players can be separated from other groups based on the results of cognitive tests results with satisfactory accuracy (61-66%). | It is possible to recognize high expertise in MOBA games using cognitive tests, but including neural correlates significantly enhances the recognition accuracy. |
| Wu at al. [41] | FPS players vs non-players (19 vs 17) | - Feature search  - conjunction search  - central and (or) peripheral search with (without) identification task | The FPS players were faster in both feature search and conjunction search. The FPS players also were faster and more accurate in the peripheral search and identification task during the central search. | Playing action video games develops a better target template to guide search in a top-down manner and improves the top-down guidance of attention to possible target locations |
| Castel at al. [42] | Video game players and non-players (10 vs 10) | - Inhibition of return  - visual search | Video game players had faster reaction times to detect targets and faster response times for visual search | Video game players rely on similar types of visual processing strategies but possess faster stimulus-response mappings in  visual attention tasks |

**List of abbreviations:**

EEG - electroencephalograml

FPS - first-person shooter

MOBA - multiplayer online battle arena

RTS - real-time strategy
