# Supplementary figures and images for: Biomarkers of professional cybersportsmen: Event related potentials and cognitive tests study
Source: PLoS One. 2023 Aug 1;18(8):e0289293. doi: 10.1371/journal.pone.0289293 (PMC10393144; doi:10.1371/journal.pone.0289293)

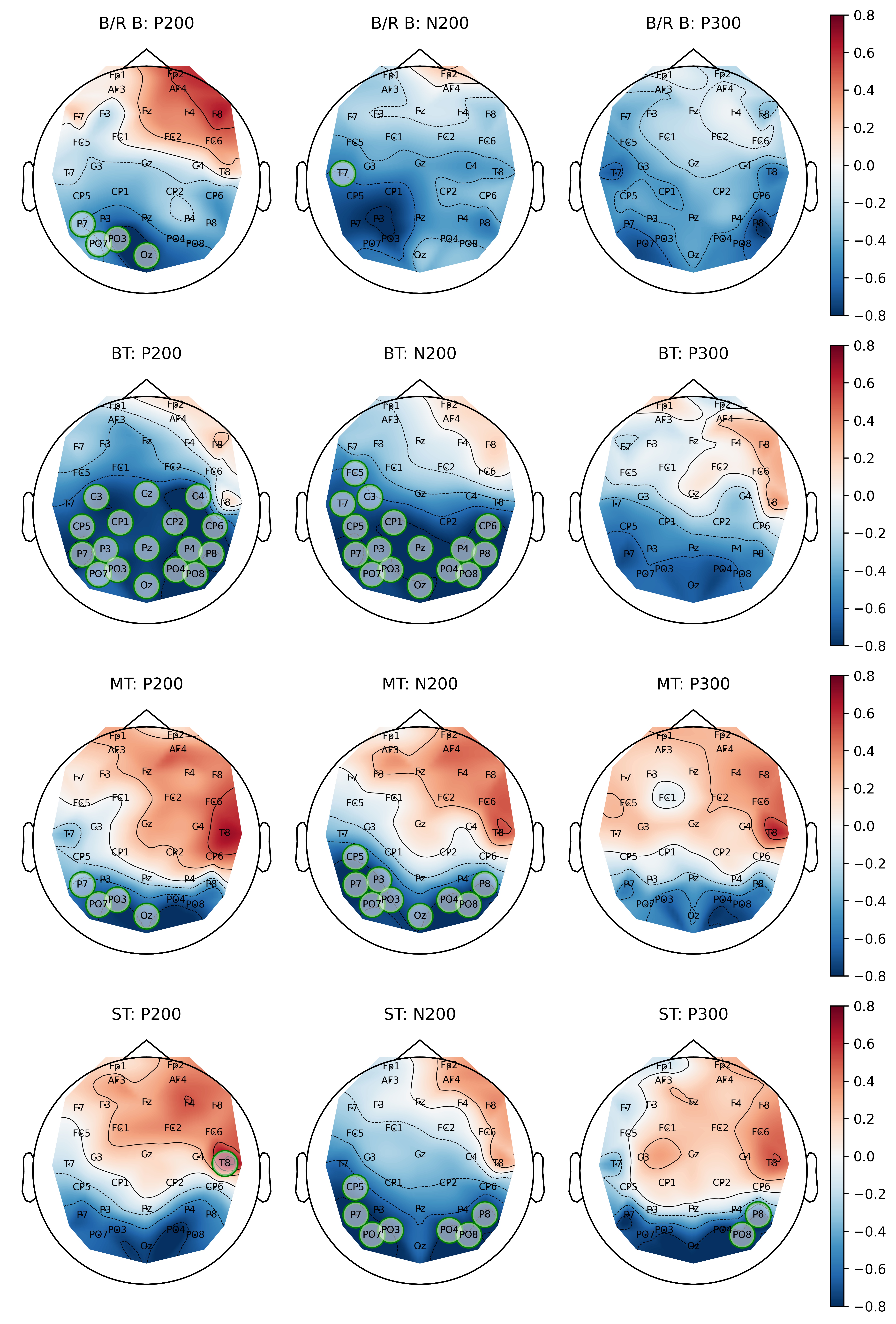

Supplement: S1 Data — (ZIP) [file pone.0289293.s007.zip › Both experiments/topomaps_non_correct_RTD_amp.jpg]

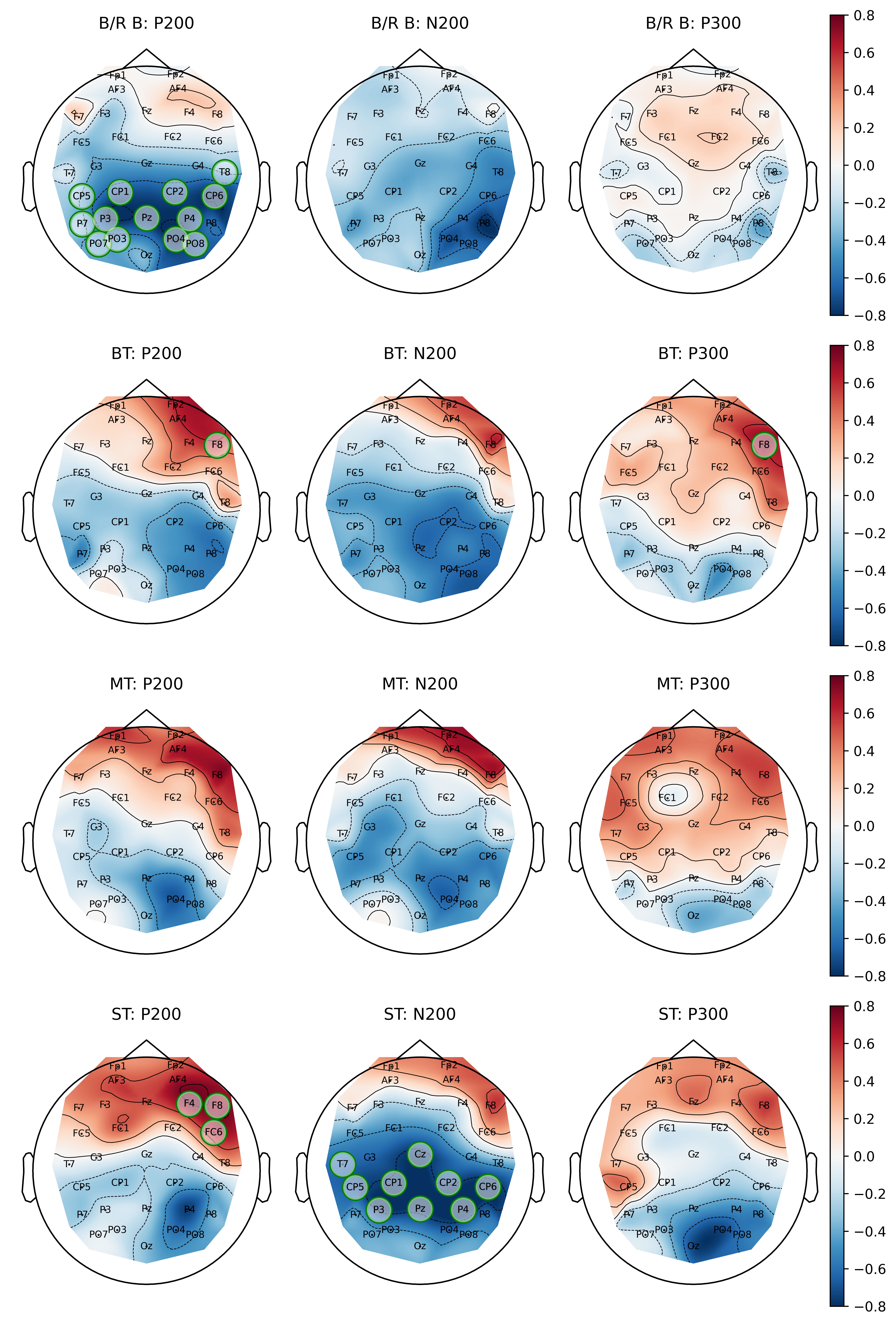

Supplement: S1 Data — (ZIP) [file pone.0289293.s007.zip › Both experiments/topomaps_non_final_VS_amp.jpg]

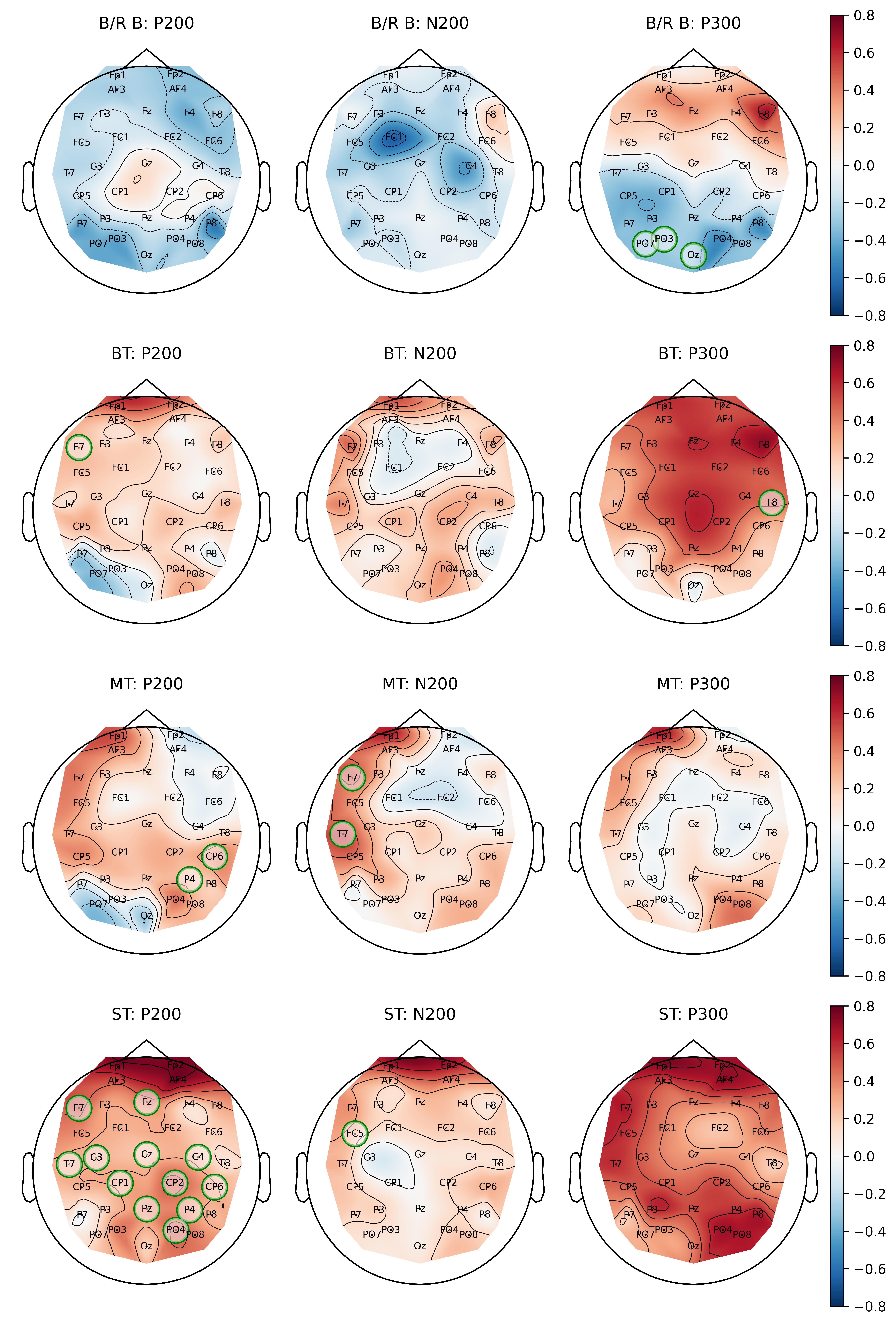

Supplement: S1 Data — (ZIP) [file pone.0289293.s007.zip › Both experiments/topomaps_pro_correct_RTD_amp.jpg]

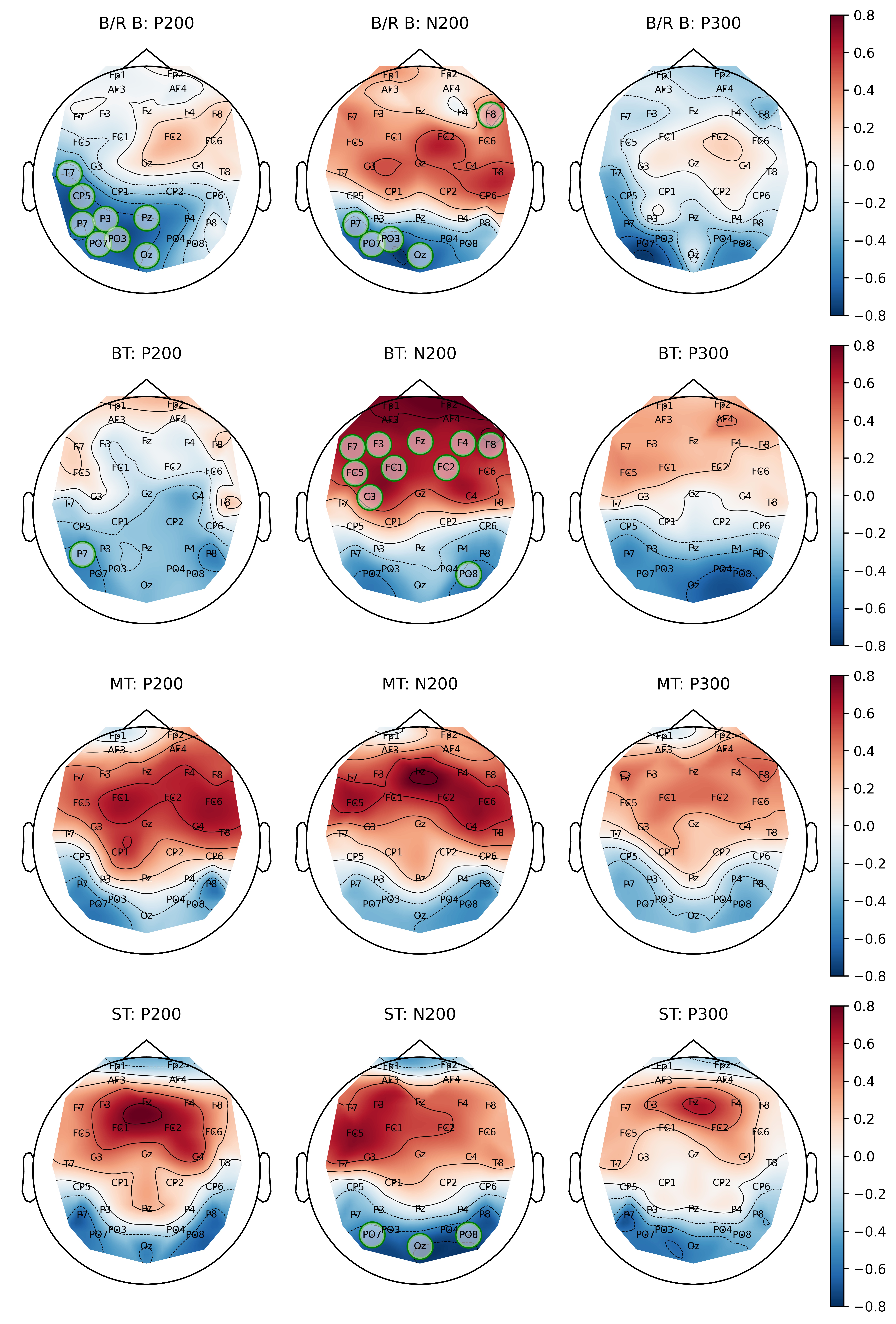

Supplement: S1 Data — (ZIP) [file pone.0289293.s007.zip › Both experiments/topomaps_pro_final_VS_amp.jpg]

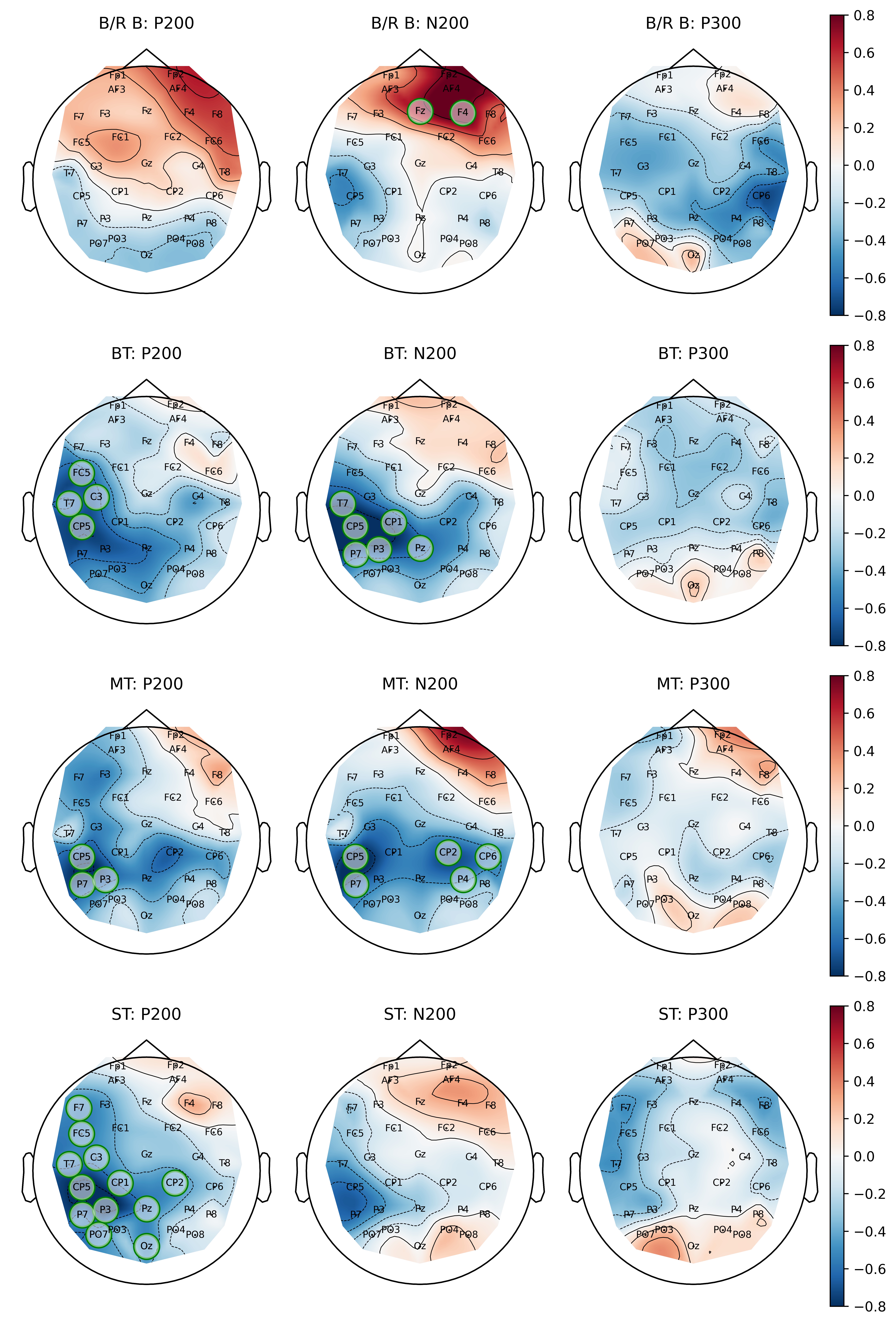

Supplement: S1 Data — (ZIP) [file pone.0289293.s007.zip › Both experiments/topomaps_pro_hours_amp.jpg]

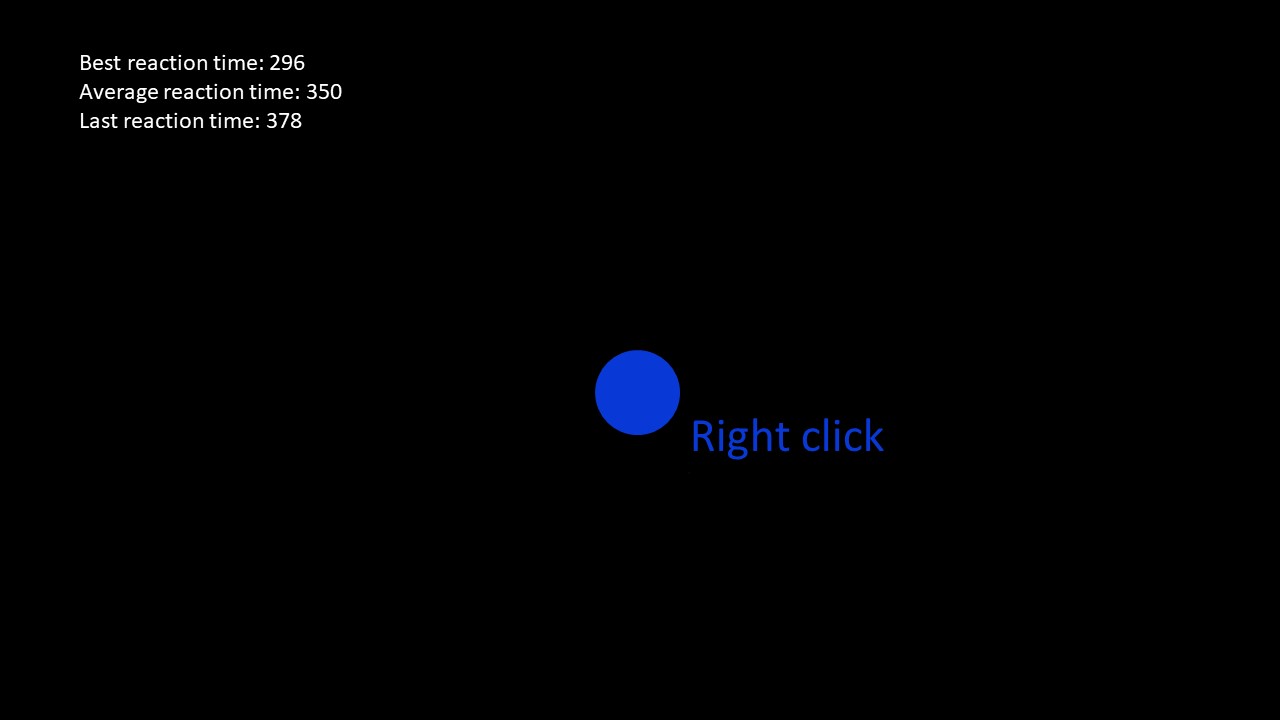

Supplement: S1 Data — (ZIP) [file pone.0289293.s007.zip › Cognitive tests/RTD_test.jpg]

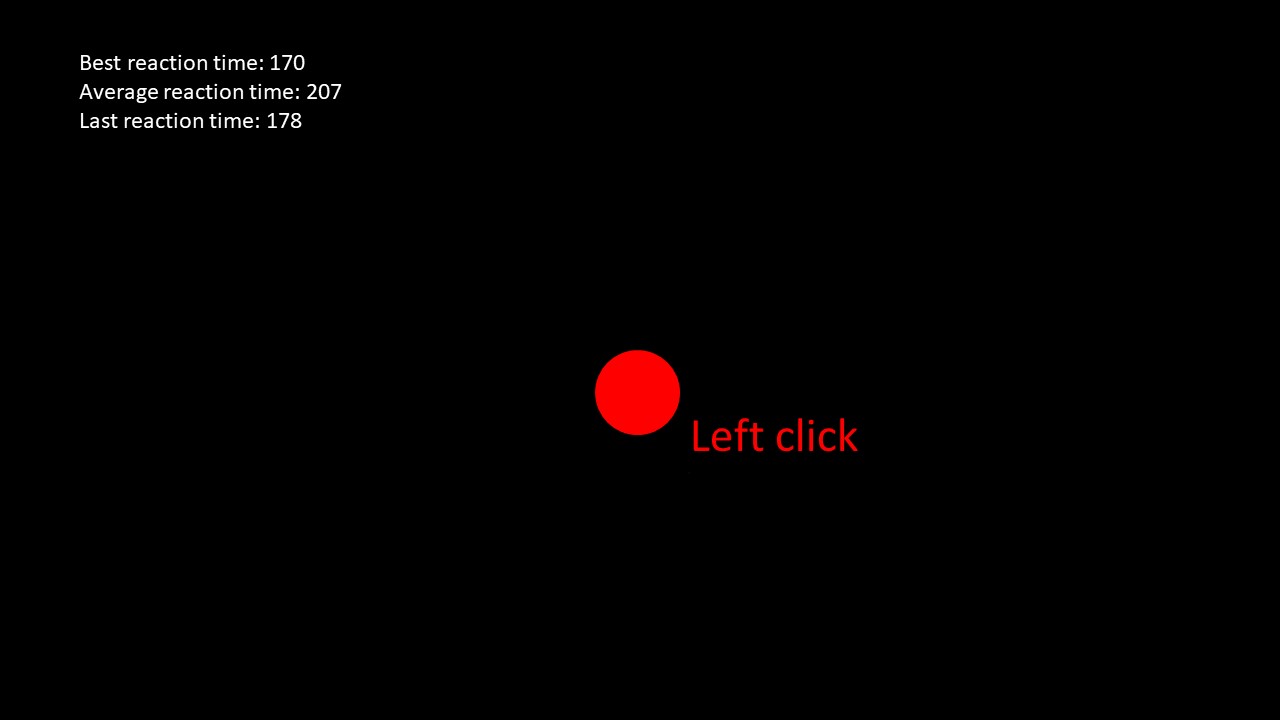

Supplement: S1 Data — (ZIP) [file pone.0289293.s007.zip › Cognitive tests/RTM_test.jpg]

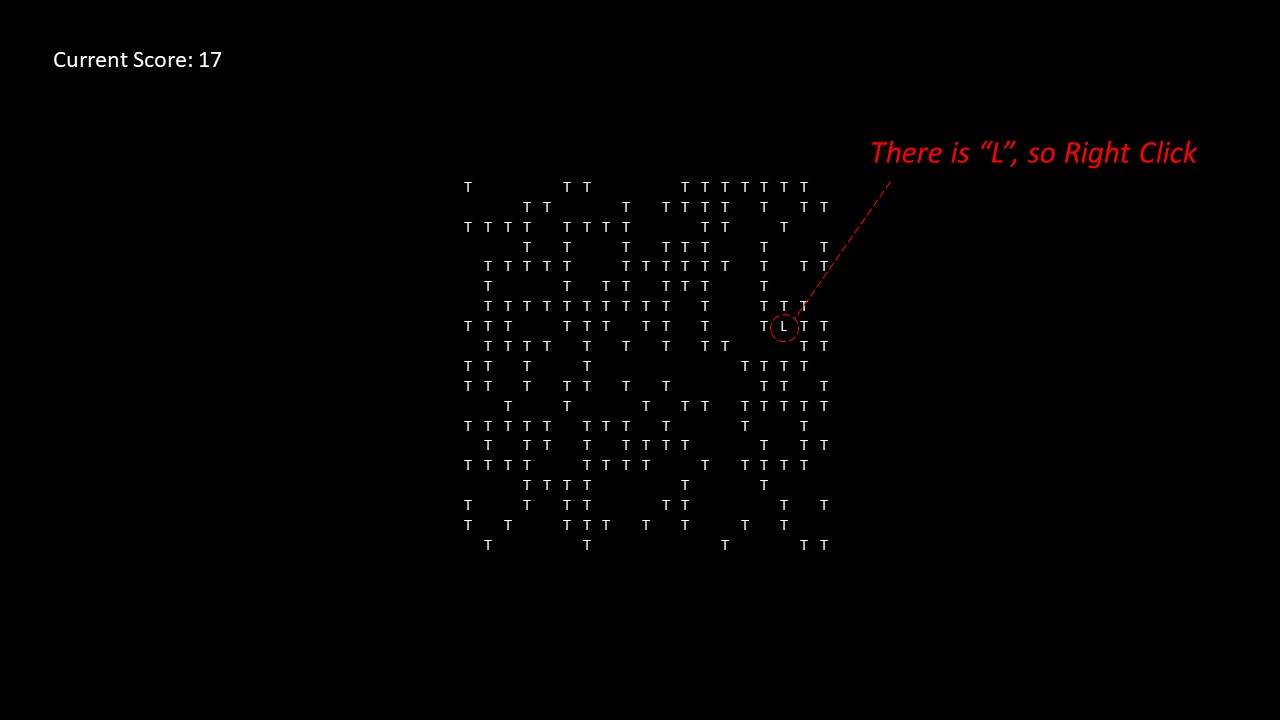

Supplement: S1 Data — (ZIP) [file pone.0289293.s007.zip › Cognitive tests/VS.jpg]

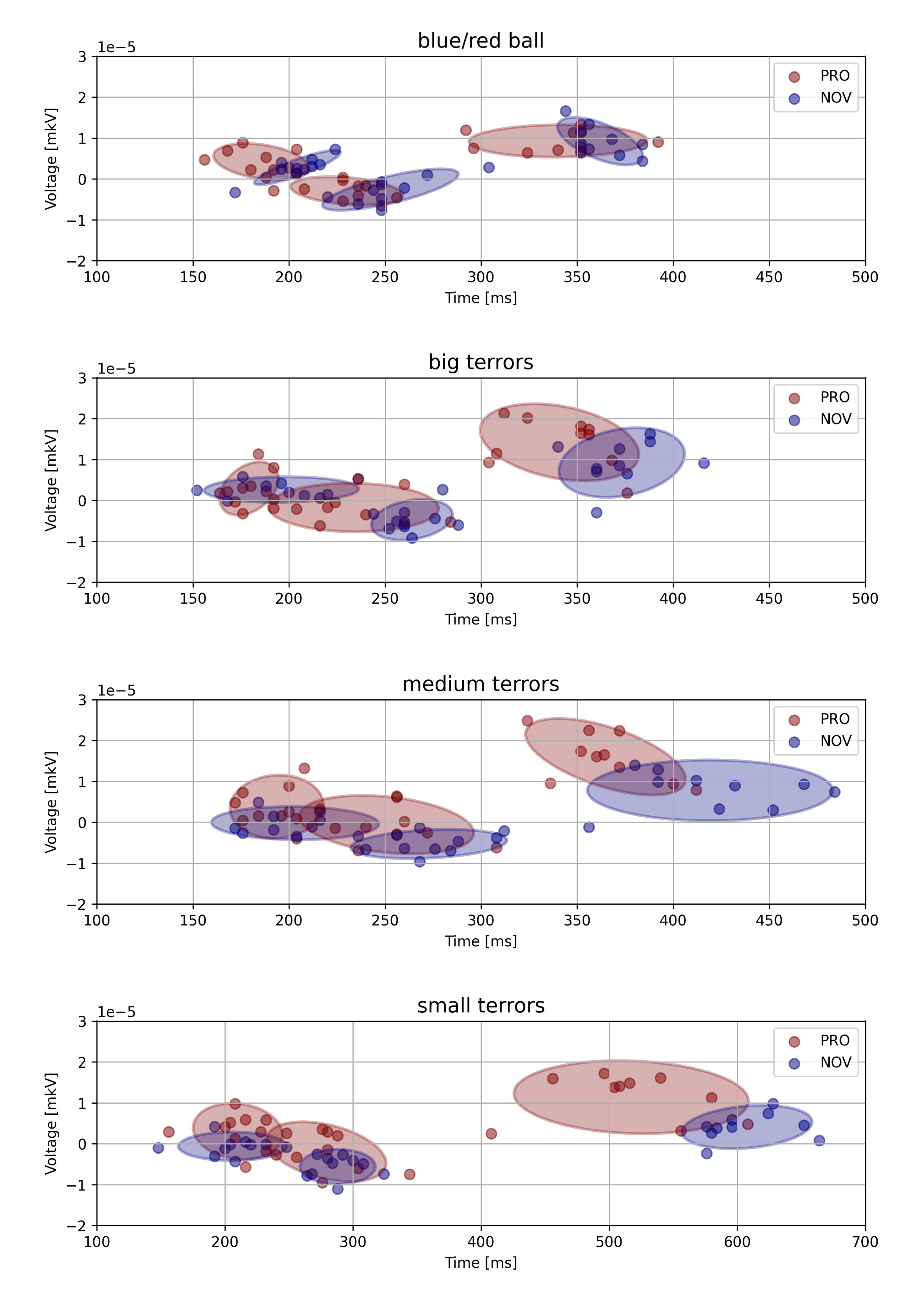

Supplement: S1 Data — (ZIP) [file pone.0289293.s007.zip › EEG experiments/ERP_compare.png]

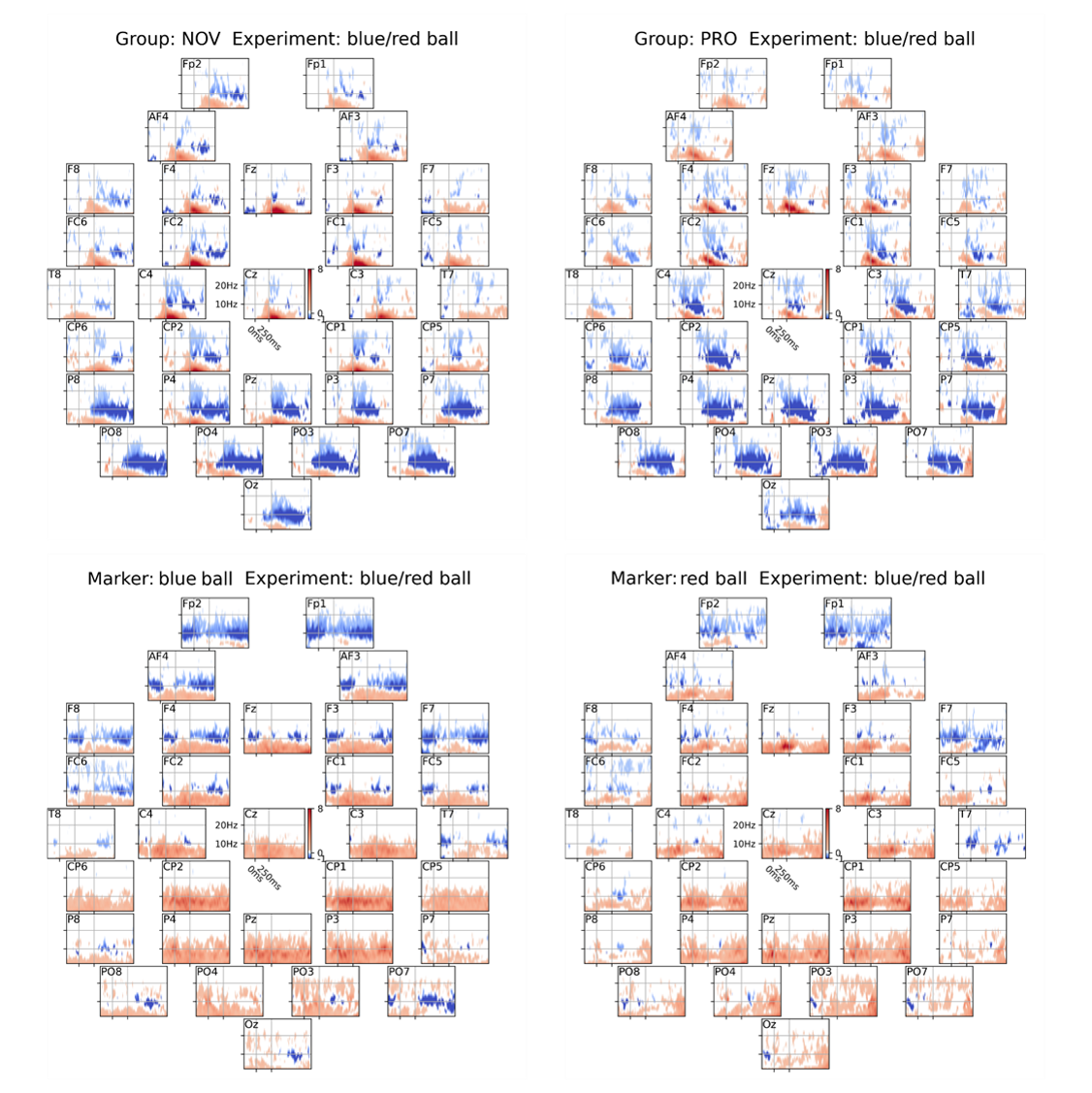

Supplement: S1 Data — (ZIP) [file pone.0289293.s007.zip › EEG experiments/SPECTR__C_1_compare.png]

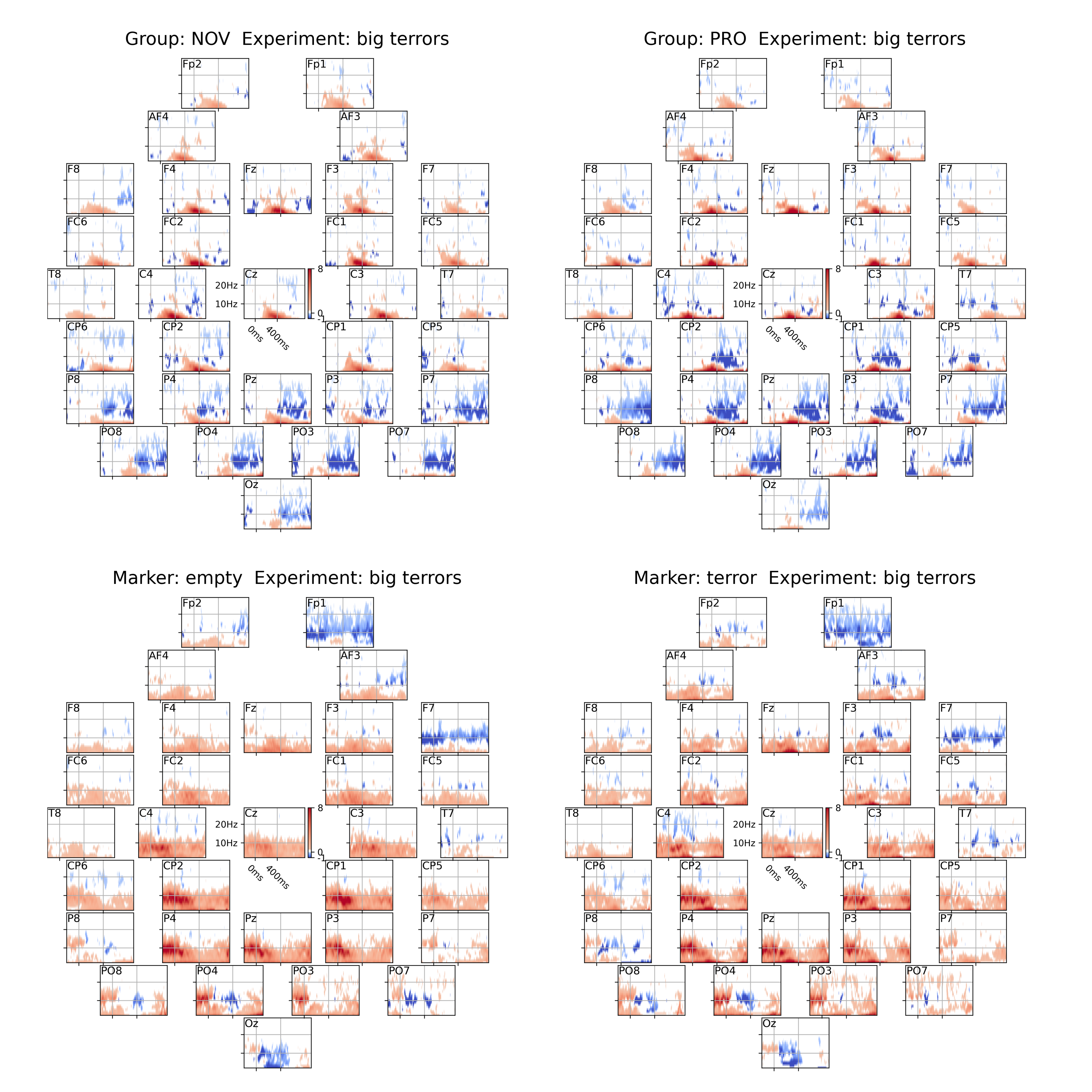

Supplement: S1 Data — (ZIP) [file pone.0289293.s007.zip › EEG experiments/SPECTR_CS_1_compare.png]

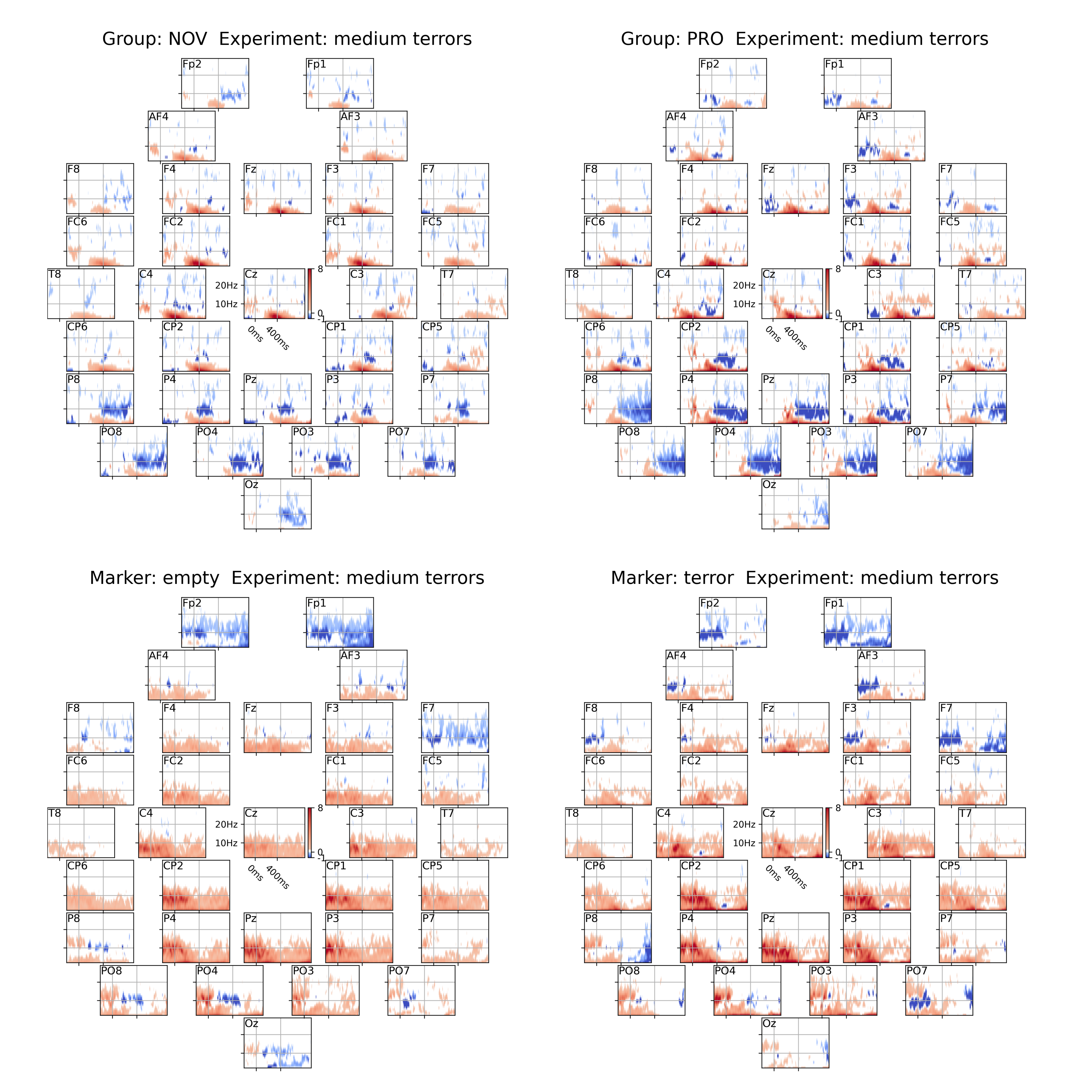

Supplement: S1 Data — (ZIP) [file pone.0289293.s007.zip › EEG experiments/SPECTR_CS_2_compare.png]

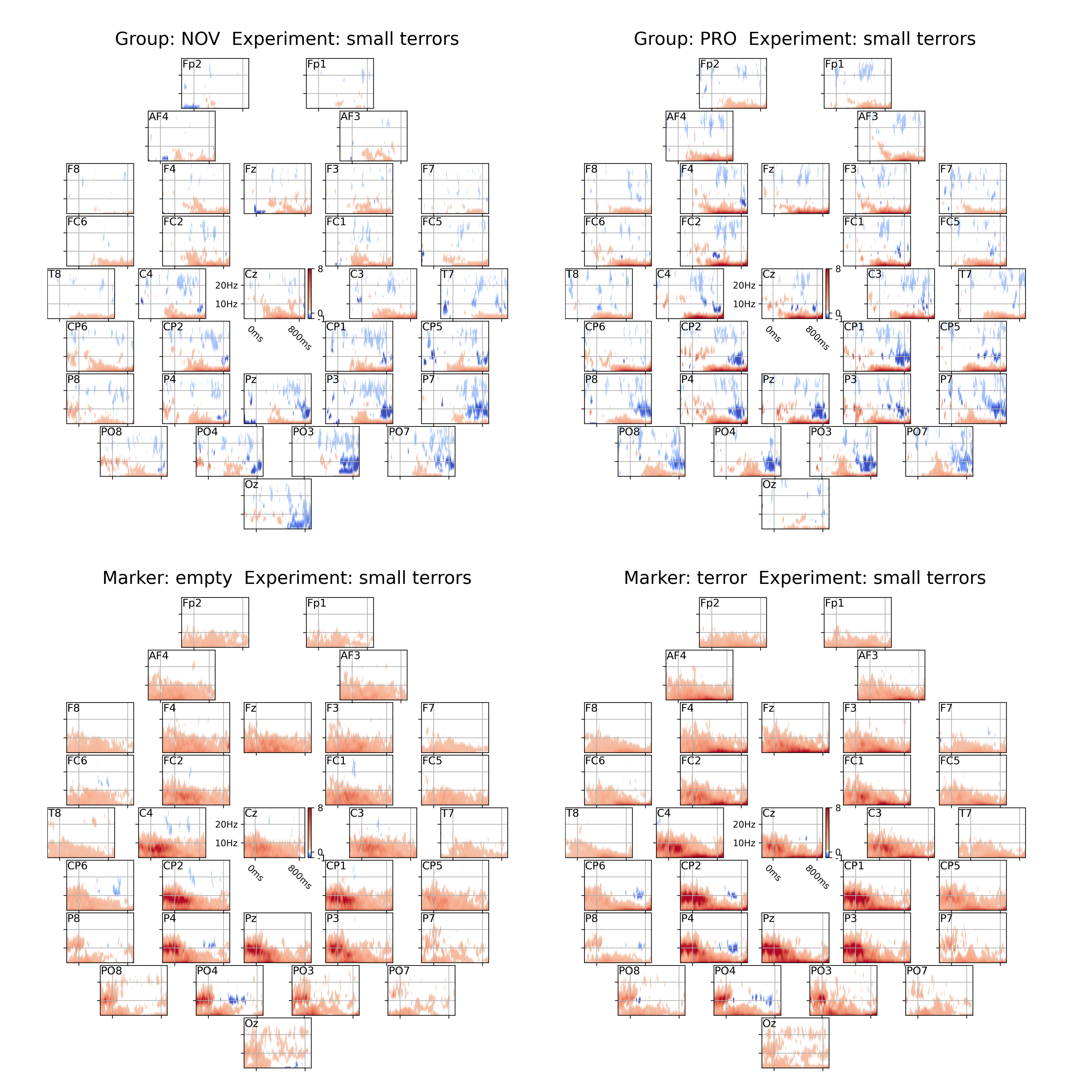

Supplement: S1 Data — (ZIP) [file pone.0289293.s007.zip › EEG experiments/SPECTR_CS_3_compare.png]
