# Supplementary material for: Biomarkers of professional cybersportsmen: Event related potentials and cognitive tests study
Source: PLoS One. 2023 Aug 1;18(8):e0289293. doi: 10.1371/journal.pone.0289293 (PMC10393144; doi:10.1371/journal.pone.0289293)
Supplement: Attachment — Submitted filename: Response to reviewers.pdf [file pone.0289293.s008.pdf]

**Respond letter to the Reviewers' comments**

**Please use the space provided to explain your answers to the questions above. You may also include additional comments for the author, including concerns about dual publication, research ethics, or publication ethics. (Please upload your review as an attachment if it exceeds 20,000 characters)**

**Reviewer #1: Authors investigated the differences in EEG biomarkers and behavioral performance between professional FPS gamers vs non-gamers. This topic is interesting and quite important in esports area.**

**Also, this paper is well-organized and provided all technique details for readers. I was wondering how ERPs could be collected if they were using commercial FPS games (e.g., counter strike here) instead of customized games. Ultimately, I understand that they made a random sequence of game screenshots of interest! This is brilliant. Also, one thing is impressive is that they used photodiode to synchronize the EEG signals and visual stimuli, which is a standard and rigorous way for ERP studies but always overlooked by other researchers.**

**For EEG data analysis, they employed EMD to calculate spectrogram instead of conventional FFT; also they investigated the specific main waves and frequencies with tensor decomposition. All these data analysis methods are the current state-of-the-art. But my suggestion is that since professional game players are well-trained people in multitasking FPS cognitive environment, so brain network-based functional connectivity analysis is perhaps better in capturing their unique brain activity patterns in comparison with non-gamers. So, this is something authors can take into consideration in their revision.**

We thank the Reviewer for throughout and positive comments.

Regarding the connectivity analysis it is been shown that due to the high signal-to-noise ratio of EEG signals and volume conduction in a head, most methods of neural connectivity analysis are not directly applicable to EEG data (e.g. the Granger Causality (GC) score, Directed Transfer Function, Partial Directed Coherence, Phase-slope Index, etc.) [1].

We thank you for your suggestion - there are interesting approaches published, for example [2] formulates functional connectivity as tensor regression problem. We plan to investigate it in depth in further research.

**In addition, I understand that authors just used a set of game screenshots in their study rather than real CS game, but I am still curious that for those non-gamers, did they experience any cybersickness or motion sickness when they watched those screenshots? If so, how cybersickness or motion sickness affected their EEG biomarkers or cognitive test performance?**

No participant in our experiments complained about cybersickness or motion sickness. We can note that several participants reported a small inconvenience due to our request to reduce the number of eye blinkings. However, breaks between the different blocks of EEG experiments were long enough to avoid any eye irritation (none was reported).

**Reviewer #2: Comments to the authors:**

**The authors aims to find the biomarkers to distinguish professional and novice video game players players. The manuscript is well written and organized. However, the resolution of all of the figures need to be improved so that the labels are readable. Moreover, some of the figures labels are missing. I have some remarks for the authors.**

We appreciate the Reviewer for encouraging comments and the suggestions. We implemented the comments of the Reviewer and hope the revised manuscript will address the Reviewer’s concerns.

We improved quality of the figures and provide missing labels in the revised manuscript.

**1. In the Section Motivation, the authors state the contributions of the manuscript are understanding the influence of eSport on a player's cognitive state and associating eSport with a real sport. Though the authors have provided sufficient results from previous studies, I suggest the authors to put all the previous findings into a table. Thus, the impact of the manuscript can be emphasized (According to the table, none of the research has use ERP to analyze FPS gamers, etc.) and the readers can have an expectation about how the metrics change (reaction time, multi-task tracking, etc.) in the following Sections.**

Thank you very much for your recommendation. We listed similar studies into two tables for analysis of EEG and Cognitive skills and present them in the supplementary materials (S1 and S2). The changes are mentioned in the main manuscript, Section for Introduction.

**2. In line 267, the authors introduce the CS:GO-related EEG experiment. During each condition, is the location of their target fixed? If the target location is fixed, the size of the target might have little effect and the visual search process might not be necessary, which changes the proposed metrics from choice reaction time to visual attention only.**

We presented three types of the game-related experiments, and only in the “Big Terrorists” experiments, the target location is fixed in the center. The size and position of the “enemies” targets approximately match the blue/red circles in the standard oddball paradigm test.

In the Medium and Small Terrorists experiments, the targets can have different sizes and do not appear at a fixed location. Thus the experiments require the participants to perform visual search.

We agree with the Reviewer that for the BT paradigm, the visual search, in essence, is not necessary. This paradigm is similar to the non-specific Blue/Red Ball (B/RB) paradigm but related to the CS:GO game.

**3. In line 325, the authors use ICA to remove blink and ECG artifacts. However, only blink removal has been explained in the following paragraph but not ECG. Further explanation needed on how the authors remove ECG artifacts.**

We thank the Reviewer for this question. ECG was removed via ICA after visual inspection of the components and according to recommendations in [3], and ICA labeling tutorial by University of California [4].

We revised the manuscript accordingly.

**4. In line 370, it seems that the sentence is not completed: on the other hand, can (?) [49] and has been…**

We are sorry for this confusion, and revised this part accordingly. The intention of this sentence is to motivate the use of multiway analysis methods while "common methods like ICA or PCA cannot take into consideration the multidimensionality".

**5. In Section ERP (line 502) and Fig. 4, it seems that the authors only plot the deviant trials. I suggest the authors to plot out standard trials as well and see if the results support the statement in line 524: the appearance timing of almost all components of professionals is approximately 1-0-40ms earlier than the novice, and the statement in line 702: Pro group has faster visual reaction.**

The plots of ERPs for standard (non-target) trials and deviant (target) trials are presented in Figure 1. The method of the ERP construction is the same as described in the manuscript. The ERPs for standard trials are much less pronounced, and some components are almost absent (e.g., P300 for all paradigms). However, one can still observe the higher amplitude and faster onset time for the PRO group during the game-specific paradigms (Big Terrors, Medium Terrors, and Small Terrors). Thus, the graph of ERPs for the non-target trials also supports the statements in lines 524 and 702. In addition, we would like to note that it is common practice to investigate only the deviant (target) trials as the ERPs are much more pronounced.

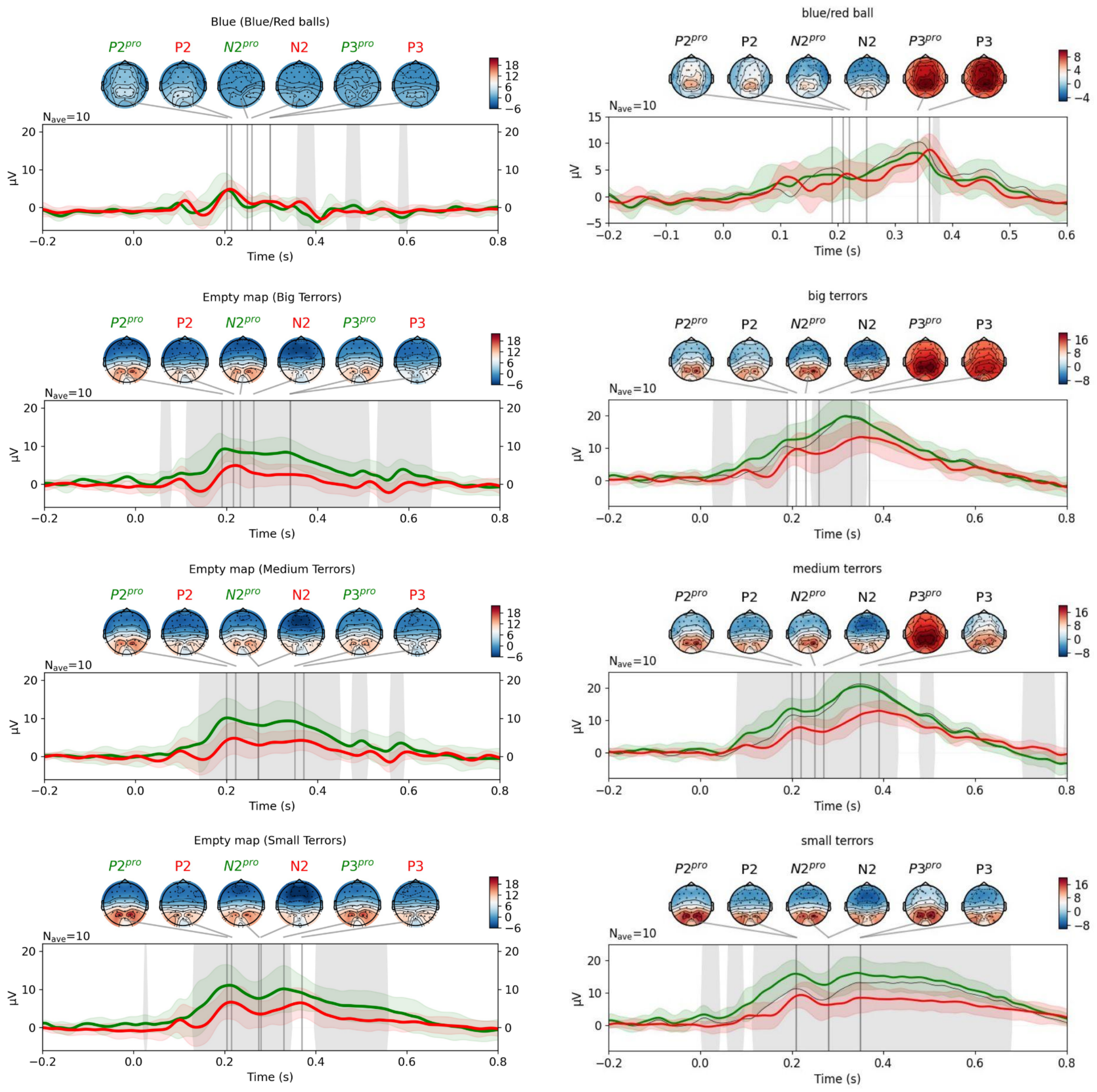


(a) (b)

Figure 1. ERP comparison of PRO and NOVICE groups for (a) standard (non-target) trials, and (b) deviant (target) trials

**6. In Fig. 5, there is an abnormally high power from 0Hz to 10Hz across the whole trials in the Pro group. One possible reason is that the Pro players are continuously moving their eye gaze to search for the targets and generating these low-frequency artifacts. Once again, this question goes back to my question 2.**

In experiments MT and ST., both groups are performing visual search. Professionals, indeed, might be faster (or otherwise more efficient) in moving the gaze during visual search. However, after the ICA blinks and eye-movement removal we did not observe any significant contributions in the frontal lobe in general or Fp1, Fp2 electrodes in particular in either ERP analysis topographic maps (Fig 4) or CPD decompositions topographic maps (Figs 6, 7, 8).

The confusion is that we did not mention the removal of eye movement artifacts in addition to blinks. We have added that to the text.

In order to avoid boundary effects which might have caused high power in low frequencies we have shortened the analyzed epochs from (-500 ms - 900 ms) to (-100 ms, 500 ms) and applied Variational Mode Decomposition (VMD) instead of EMD. Further, we have analyzed the produced IMFs to ensure their purity from artifacts.

Firstly, we can show that high power in low frequencies is not generated by eye movement using VMD. On the frequency histogram distribution (Figure 2) you can see that most of the low frequency power is occupied by the first IMF (blue).

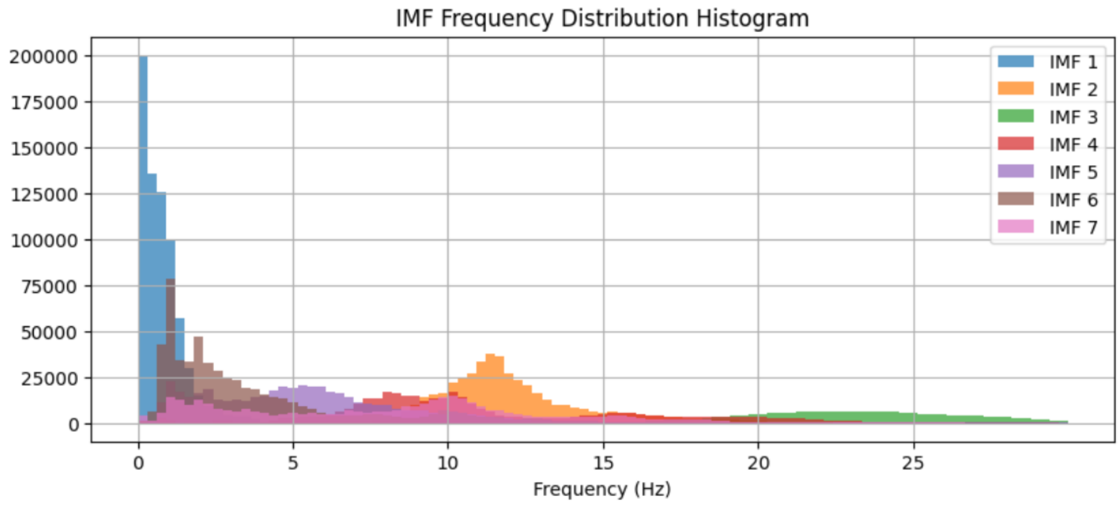


Figure 2. Frequency distribution of IMFs

Secondly, we can show that the first IMF does not contain eye artifacts. Figures 3 and 4 depict the mean topographic maps of this low-frequency IMF for each group respectively. One can see there is no substantial amplitude in the frontal section of professionals at any time. Most of the power is occupied in parieto-occipital parts - zones where we expect to see ERP. From that, we surmise there are no substantial eye movement artifacts in the collected data.

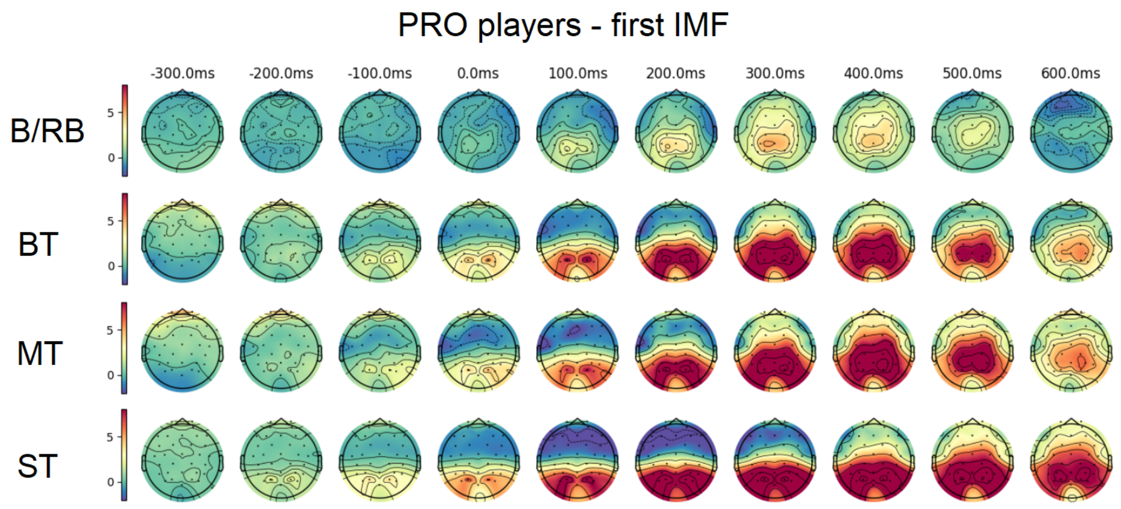


Figure 3. Topographic maps of first IMF of VMD decomposition of PRO players in each experiment

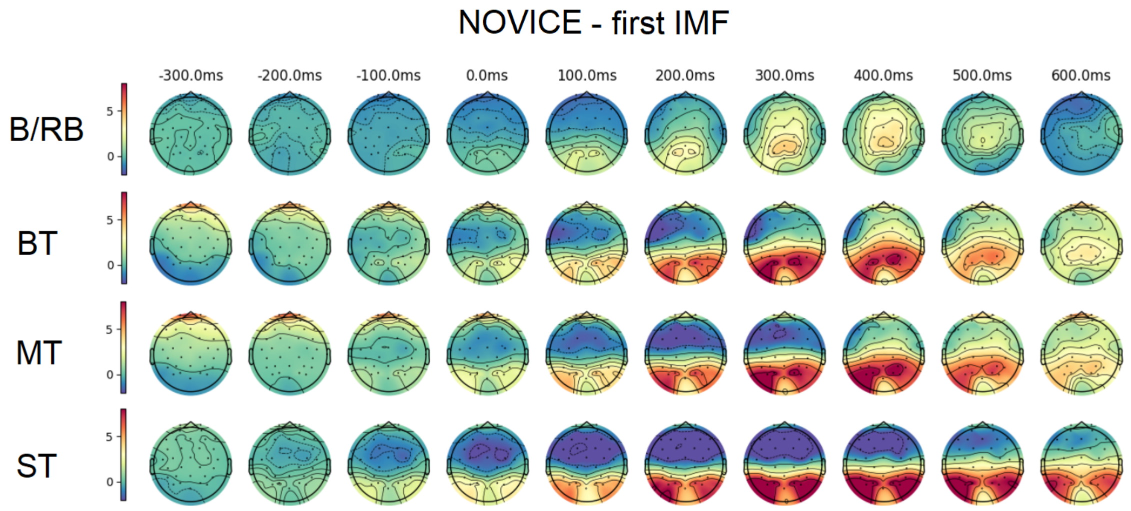


Figure 4. Topographic maps of first IMF of VMD decomposition of NOVICEs in each experiment

**7. In Fig. 6, though the authors have described the axis-labels in the caption, it will be more reader-friendly if the axis-labels are on the figure. Also, the resolution of the figure is too poor to read the legend.**

Thank you for your comment. We added labels and increased the resolution of the figure.

**8. Following my question 6 and 7, it seems that CPD component 1 might explain why there is a significant increase in low-frequency power in the Pro group. I suggest the authors to separate the trials by groups and recalculate the power once again.**

As described in the answer to question 6, we think that high power in low frequency components does not lie in the difference of eye movement patterns between groups, but in the brain activity itself.

Note that we decided to redo the CP decomposition with, cropped by time, spectrums to guarantee the absence of the boundary effect distortion. In other words, we updated the pictures of CPD for the spectrum (Spatial-Time-Spectral analysis, see Figs 7 and 8 in the manuscript). Moreover, we also included the CPD of the Spatial-Temporal tensors (see Fig 6 in the manuscript). Therefore, the further shown pictures correspond to the updated figures.

As per your suggestion, we have performed the CPD of each group separately (Figures 5 and 6). One can see that the components did not change drastically in either dimension (topographic maps, spectral, or time evolution). Moreover, decomposing groups together allows us to compare them not only qualitatively (via visual inspection of selected components) but also quantitatively (via statistical tests over component power or prominence in each group).

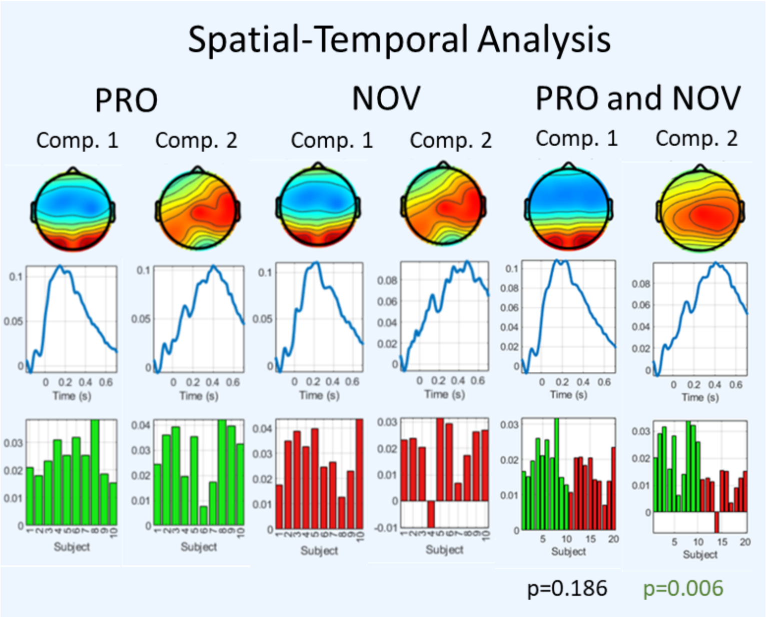


Figure 5. CPD of Professionals (left), Novice (middle), and both (right) groups during spatial-temporal analysis for the Small terrors EEG paradigm. The detailed explanation can be found in the main paper text, Fig 6.

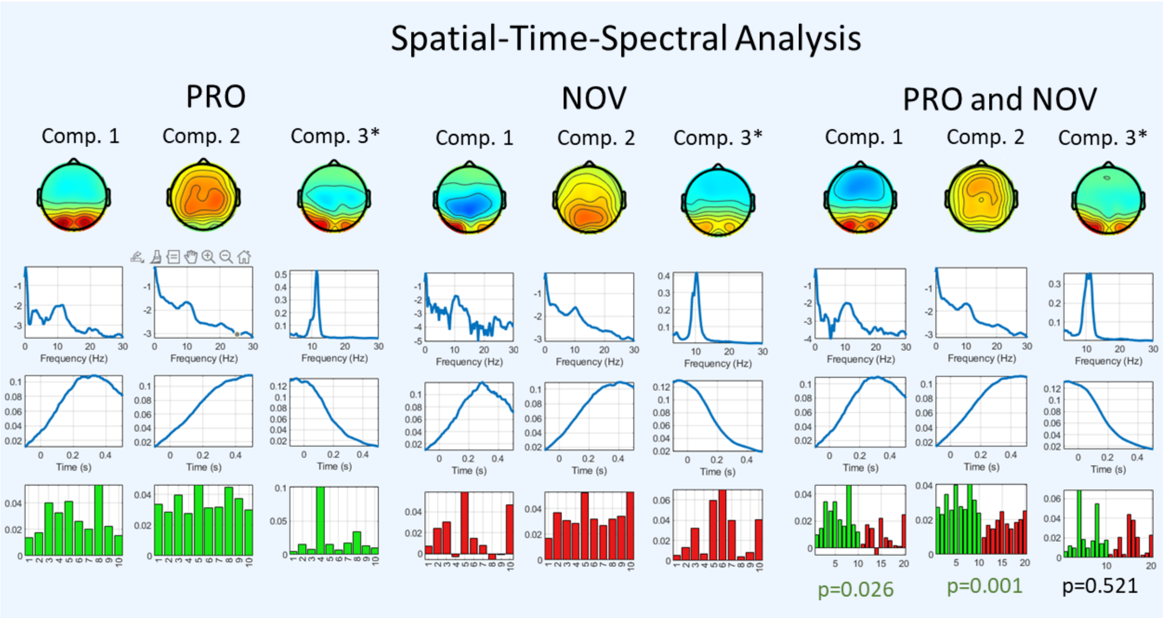


Figure 7. CPD of Professionals (left), Novice (middle), and both (right) groups during spatial-time-spectral analysis for the Small terrors EEG paradigm. The detailed explanation can be found in the updated main paper text, Fig 7 and 8.
* 3rd component corresponds to CPD for frequency band tensor (see Fig 8 in the updated manuscript).

**9. In Fig. 6, component 2 has response right before stimuli onset. Does it mean subjects expect there is an incoming stimuli and begin to prepare for the potential deviant trial? Are the component 2's activities same between two groups? Also, does CPD include only deviant trials or all the trials? The statement in line 732 is potentially weaken by these questions.**

No, the onset in component 2 after 500ms is not due to anticipation of the next pictures but because of overlapping with the next stimulus. Due to simplicity, the B/RB paradigm is shorter (stimulus: 0.25s, blank: 0.5s) than the others (stimulus: 0.4-0.8s; blank: 1s). The information about it is in Table 1 of the manuscript. Despite the different timing of the paradigms, we decided to make time axis limits to be the same for all paradigms in Fig. 6 for easier visual comparison between them. To avoid such confusion, we have cut curves of the CPD components for the BR/B experiment representation.

**10. Out of my personal curiosity, why there is no EEG recordings during cognitive tests? I expect there is a difference in the baseline before stimuli onset between Pro and novice players.**

We were limited by time already generously provided by the professional eSport team, and since the cognitive tests took more than the ERP already, burdening them with full 32 electrodes gel application was out of the question. We did record 8-channel dry electrodes though. Unfortunately, movement artifacts were too pronounced to use it for any ERP analysis.

**Some minor suggestions:**

**1. In line 41, EMG was used without definition. I suggest the authors to add it in line 39: [2] investigated electromyography (EMG), the electrical activity of muscle tissue, of lower....**

Corrected

**2. In line 57, EGG was used without definition. I assume it is a typo for EEG.**

Corrected

**3. In line 205 and line 220, the authors seem to make the same claim while referring to different references (6 in 205 and 4 in 220).**

We used the references on two different review papers 4 and 6. They all are appropriate to line 220 (old version), so now we use both references.

**4. In line 433, PLS and CCA were used without definitions.**

The definitions of PLS and CCA were added

**5. In line 720, Gf was used without definition.**

Gf is related to the fluid intelligence, corrected

References


1. S. Haufe, V. Nikulin, and G. Nolte. Identifying brain effective connectivity patterns from eeg: performance of granger causality, dtf, pdc and psi on simulated data. BMC Neuroscience 2011 12:1, 12:1–2, 7 2011
2. E. Karahan, P. A. Rojas-Lopez, M. L. Bringas-Vega, P. A. Valdes-Hernandez, and P. A. Valdes-Sosa. Tensor analysis and fusion of multimodal brain images. Proceedings of the IEEE, 103:1531–1559, 9 2015
3. Iriarte J, Urrestarazu E, Valencia M, Alegre M, Malanda A, Viteri C, Artieda J. Independent component analysis as a tool to eliminate artifacts in EEG: a quantitative study. J Clin Neurophysiol. 2003 Jul-Aug;20(4):249-57
4. https://labeling.ucsd.edu/tutorial
