# Supplementary material for: Biomarkers of professional cybersportsmen: Event related potentials and cognitive tests study
Source: PLoS One. 2023 Aug 1;18(8):e0289293. doi: 10.1371/journal.pone.0289293 (PMC10393144; doi:10.1371/journal.pone.0289293)
Supplement: Attachment — Submitted filename: Response to reviewers.pdf [file pone.0289293.s009.pdf]

Respond letter to the Reviewers' comments

4. Have the authors made all data underlying the findings in their manuscript fully available?

The PLOS Data policy requires authors to make all data underlying the findings described in their manuscript fully available without restriction, with rare exception (please refer to the Data Availability Statement in the manuscript PDF file). The data should be provided as part of the manuscript or its supporting information, or deposited to a public repository. For example, in addition to summary statistics, the data points behind means, medians and variance measures should be available. If there are restrictions on publicly sharing data—e.g. participant privacy or use of data from a third party—those must be specified.
Reviewer #2: No
Reviewer #3: No

Thank the Reviewers for paying attention to this aspect, we will provide the data underlying the findings via the cloud file storage: https://disk.yandex.ru/d/jgW45J3tJKUAWw

6. Review Comments to the Author

Please use the space provided to explain your answers to the questions above. You may also include additional comments for the author, including concerns about dual publication, research ethics, or publication ethics. (Please upload your review as an attachment if it exceeds 20,000 characters)

Reviewer #2: I appreciate the authors' efforts. Most of my questions have been properly addressed. One last thing, I strongly recommend the author, for reference [4] ICLabel, to cite the paper instead of website. The paper title is "ICLabel: An automated electroencephalographic independent component classifier, dataset, and website".

Thank you for the remark, we cited the suggested reference at a proper place.

Reviewer #3: In this study, the authors investigated the differences in EEG and cognitive test performance between eSport PRO and NOVICE players. The results showed that PRO players had significantly faster reaction times and stronger and earlier event-related potentials (ERPs), as well as stronger pre-stimulus alpha power, compared to NOVICE players. Visual search performance, however, did not differ significantly between the two groups. Using tensor decomposition analysis, the authors found that the second component of the p300-like component was significantly stronger in PRO than NOVICE.

Furthermore, correlation analysis revealed different patterns between PRO and NOVICE players, with PRO players showing higher correlations between ERP features in CS:GO-related paradigms, while NOVICE players did not.
Overall, this study provides valuable insights into the neural and cognitive correlates of eSports professionals. The authors thoroughly explored potential factors that may differ between PRO and NOVICE players. However, there are a few major conceptual and analysis issues, as well as additional clarity, that need to be addressed before this study can reach the required bar for influencing thinking in the field. Nonetheless, the study's findings are interesting and contribute to the ongoing research on eSports performance.

General comments
1. The authors presented a comprehensive analysis of the EEG and cognitive test performance differences between eSport PRO and NOVICE players. To improve the clarity of the paper, it would be beneficial for the authors to prioritize the most significant findings and clearly link them to the hypotheses listed in the Introduction.

Thank you for your recommendation. We made explicit conclusions about the hypotheses listed in the Introduction and in the Conclusion section.

2. Furthermore, some of the conclusions drawn by the authors appear to be unsupported or even contradicted by the empirical findings. It is important for the authors to carefully examine their results and revise their conclusions accordingly. Additionally, they should aim to provide more clarity in describing and interpreting their results.

We revised the related part to address this important comment.

3. One area that could be further developed is the discussion of the cognitive functions associated with the brain components investigated in this study, such as the ERP P200, P300, N200, and alpha-band oscillations. It would also be helpful for the authors to discuss how these components are related to each other and to the cognitive demands of eSport gameplay.

Thank you for the suggestion, we have added the cognitive functions related to ERPs

Regarding the relation of ERP components and the esport (or other sport) demands, we have presented an overview table or related research and observed differences in groups (or conditions in one group) in the supporting information S1.

Detailed comments

Abstract: do the authors intend to say pre-stimulus alpha instead of post-stimulus alpha? In Figure 5 and corresponding main text from Line 561, as well as Discussion described the effects otherwise.

Corrected.

Line 53: It is better to spell out what are some early/late components and which ones do authors focus on and why.

We revised the text according to this comment. Specifically for line 53, the components were P100, N100, P200, and N200.

Line 79 & Line 571: define the frequency range of theta, alpha and beta

We added these frequency ranges, Alpha (8-12 Hz), beta (13-30 Hz), theta (4-8 Hz), in the manuscript text.

Line 118: "faster" in what aspect?

"faster" means a shorter reaction time. We have rephrased this sentence.

Line 180: it's unclear about what "representation" means in this context. Do the authors mean the underlying neural processes of a certain cognitive function?

The idea is that the brain activity during the EEG experiments represents some cognitive functions or characteristics. For our case, the functions (characteristics) are reaction time, visual search, and decision making. That is why we conducted the corresponding cognitive tests. Thus, the "representation" of the brain activity means cognitive functions involved in this brain activity.
We have rewritten the corresponding part to make it more clear.

Line 227: please provide more context to motivate EMD analysis

As for line 227, the main idea was that we studied spectral and temporal domains. The spectral method (Empirical Mode Decomposition) is not crucial for the hypothesis mentioned in this line. However, we have added to the text (in subsection "ERPs and Spectrograms of Section "Materials and Methods" subsection "ERPs and Spectrograms") additional motivation for the usage of VMD.

Line 251: for a more balanced literature review, please also provide counter-evidence of the benefits of eSports if there is any

Correted, added citation to the Introduction section.

Line 234: were the CS:GO screenshots directly extracted from the game? Plus that PROs brought their own equipment. One might argue that the superior performances (e.g. faster reaction times) of the PRO may be due to familiarity of the scene and better equipment.

Reaction time measured in cognitive experiments were not game-related, thus cannot be attributed to familiarity.
The equipment provided for novices matched all important parameters of professional equipment (connection speed, optical resolution, ergonomics, etc.). However, since the professionals spend quite some time using their own equipment and are adjusted to it (e.g. keystroke bounce, surface friction) and claim to be sensitive to such nuances we decided to allow them the usage of their own equipment.

Line 397: please be more clear about why ICA/PCA cannot take into consideration of multi-dimensional data.

ICA/PCA were designed to work with matrix form data, so they can not be applied directly to tensors. We updated the corresponding part in the main text. Please, refer to citation (Cichocki A, Mandic D, Lathauwer LD, Zhou G, Zhao Q, Caiafa C, et al. Tensor decompositions for signal processing applications: From two-way to multiway component analysis. IEEE Signal Processing Magazine. 2015;32:145–163. doi:10.1109/MSP.2013.2297439)

ICA and PCA are methods that decompose a matrix **Y** into a product of two matrices **A** and **X: Y = A X**
where **Y** represents observed data samples, **A** represents a mixing matrix, and **X** represents latent components.
ICA and PCA have different assumptions and objectives for finding **X**. ICA assumes that **X** has (statistically) independent rows, while PCA tries to find **X** that maximizes the variance of **Y**. Both methods result in (almost) orthogonal rows of **X**.

ICA and PCA are not suitable for data that has more than two dimensions, such as a tensor **Y** which can have multiple modes, such as channels, time frames, trials, frequency bins, or subjects. Applying ICA or PCA to a tensor **Y** would require either slicing or unfolding **Y** along one mode, which would lose information about the interactions between modes or the commonality across slices.

A better way to decompose a tensor **Y** is to use a method that can capture the simultaneous interactions between modes and the common latent components across slices. Such a method would find a set of matrices **A**1, **A**2, ..., **A**n and a core tensor **G** that satisfy **Y** = **G** x1 **A**1 x2 **A**2 x ... xn **A**n. This is called a tensor decomposition.

Line 426: it would be beneficial for the authors to have a summary table here including all result parameters from different CT and EEG tests.

Thank you for the suggestion. We added Table 2 which contains all considered parameters related to cognitive abilities. We also updated the corresponding text section.

Line 452: what tasks did participants do in the reaction time tests?

The full description of the cognitive tests including the participants' task is presented in the "S3 Appendix. Cognitive tests description" as was mentioned in the last paragraph of the subsection "Experiments procedure" (section "Materials and Methods").

We agree that it makes sense to add one more link to this appendix near the mentioned part of the text. Therefore, we did it near the table described in your previous comment.
As for the task during the reaction time tests: for the RTM and RTK tests, the participant looks at the black screen and should click the mouse (for RTM) or keystroke the space button (for RTK) when he/she sees the red circle. During the RTD test, the participant should left-click if the red circle and right-click if the blue circle.

Line 457: how was the threshold determined?

We determined the threshold values (limits) based on the physiological and psychological limits of human reaction time. We assumed that any reaction time longer than 500 ms for the RTM (and RTK) test (on simple reaction time) or 700 ms for the RTD test (on choice reaction time or decision making) indicated a lack of concentration or attention from the participant. Similarly, any reaction time shorter than 120 ms for the RTM (and RTD) test or 140 ms for the RTD test suggested a random guess rather than a genuine response. These values are consistent with the literature on human reaction time (Deary IJ, Liewald D, Nissan J. A free, easy-to-use, computer-based simple and four-choice reaction time programme: the Deary-Liewald reaction time task. Behav Res Methods. 2011 Mar; 43(1):258-68. doi: 10.3758/s13428-010-0024-1. PMID: 21287123.).

We also verified that these thresholds did not affect the distribution of reaction times by examining the results of the cognitive test (see Fig. 2). After applying these preliminary

filters to remove unrealistic and inadequate values, we further eliminated outliers based on the 1.5 interquartile range (IQR) criterion.

We added the reference to the mentioned research to the main text.

Line 469: incomplete sentence
Corrected.

Line 504: "In other words, it obviously implies that professional players have a faster reaction time than novice players." is redundant
We have deleted this sentence.

Fig 2: in my view of the figure, there were no abcde labeled. Please check for all other figures too. In addition, please add the units for all the subplots for this figure and all other figures.

In the form available to us, all abcde labels are present. We made the size of these labels large. Besides, we added the units where it is possible. Some metrics, like correlation or probability density, do not have units.

Table 3 and corresponding statistics in the main text: it would be important to add the effect size since p-value is very easily biased by the number of the datapoints. Is there a reason why only showed ST but not BT and MT conditions?

We added the values of the effect sizes to the table and updated the corresponding parts in the main text.

The main reason to show only ST is that the Count error for BT and MT are approximately equal to zero (mostly all participants counted the number of target stimuli correctly). However, we decided to add the value for MT to the show results as both MT and ST require a visual search. For these EEG paradigms, a terror could appear in different parts of the shown picture. In contrast to this, a terror appeared only at the center part of the picture during the BT paradigm. In addition, we updated the corresponding parts of the text.

Fig 3: (c) (d) labels were inaccurate – should be RTM & RTD
Corrected

Line 527: the authors mentioned that VS test was not related to CS:GO game directly. Was the RT tests related to CS:GO directly?

No, similar to VS test, the RT tests were not directly related to CS:GO. They are non-specific tests on reaction time. We updated the manuscript by mentioning this explicitly in the subsection “Experiments procedure” (section “Materials and Methods”).

Fig 4: How were the channels selected? Make sure the y-axis scale is the same.

Channels were selected from the centro-occipital region, where the P300 component of ERP was most pronounced (this can be seen on each topographical map on the figure 4).

The y-axis scale was the same for the game-specific paradigms (BT, MT, and ST). For the B/RB paradigm, the y-axis scale was made larger on purpose for easier perception.

Line 541: the word “latency” and “onset time” were used interchangeably. I suggest use consistent terms throughout the paper.

Thanks for the comment. We updated the text and left only “latency”.

Table 4: why only showing Pz instead of the average as in the Figure 4?

We have updated the table by showing the metrics of ERPs calculated as in Figure 4. We also have updated the corresponding text in Results and Discussion sections.

Line 578: how “high” is the correlation? Please be clear about the cut-off points for selecting components.

“Correlation” corrected to “consistency across experimental paradigms”. We updated the corresponding part of the text.

Fig 6: incomplete sentence “All paradigms were grouped the number of the component.”

Corrected.

Line 610: what is the third component?

“The third component” meant the selected component of the decomposition for a high-frequency band. We rephrased this sentence to be more accurate and clear.

Line 612: suggest consistent terms such as “occipital“ instead of “back part of the head”
Done

Line 625: 3 to 30 Hz?
Yes, corrected!

Line 642: "CS:GO-related paradigms are paradigms are emphasized from the non-specific paradigm" meaning "correlations are higher in CS:GO related paradigms"? Please rephrase to be clearer.
Rephrased.

Line 656: "this correlation does not pass the 5% threshold of p-value" can simply be rewritten as "not significant"
Corrected

Line 665: it would be awesome if the authors could highlight the ones based on those two criteria

All of the mentioned in the manuscript significant correlations and presented figures (11-12) are based on these two criteria. We rephrased the corresponding subsections to make it more clear.

Fig 11 -13: please interpret why the effects are stronger in these certain channel locations in Discussion.

We have added our interpretation at the end of the subsections "Relations between Cognitive tests and ERP signals" and "Correspondence of player`s level to the cognitive tests and ERPs" in the Discussion.

Line 708: one general comment on the writing style of the paper is that it would be more informative to report the direction of the effect rather than simply stating that there is a difference between PRO and NOVICE players. The authors could provide more clarity by specifying whether the effect is stronger or weaker in one group compared to the other.

Thank you for this comment. We reread the manuscript and updated the text by adding the effect direction.

Line 721: please consider rephrasing this sentence to more clearly contrast which correlations are higher in PRO vs. NOVICE

This part is dedicated to intragroup analyses. We rephrased the corresponding part of the text to make it more clear.

Line 726: meaning fewer "significant" correlation?

No, the mentioned correlation is not significant. This is not statistically correct to rely on the non-significant values, so we decided to exclude this from the text. The corresponding part of the text was updated together with solving the previous comment.

Line 730: the finding of faster mouse clicking could be due to the equipment that PROs brought with them. It would be helpful to know the baseline clicking rates of PRO and NOVICE.

We provided a game-level computer mouse for all of the participants. Since the professional players usually have a habit of using their mouse, we allowed them to use it. The majority of them did it. The novice players could bring their own devices too, but they did not do it. The same is for the keyboard and mouse pad. In other words, the equipment was the same level for all of the participants, hence, the equipment is very unlikely to be the cause of the faster mouse click of the professional players.

Line 761: please expand and explain the findings of those ERP components and they can relate to each other.

Added paragraph citing findings on cognitive functions connected to ERP components.

Line 764: "PRO group does not show statistically significant differences" from NOVICE?
Corrected.

Line 768: spell out what are lower significant differences. Are these shown in Table 4?
No, we meant the significant difference only in one component, corrected.

Line 772: both pre- and post-stimulus alpha-band power were significantly stronger in PRO than NOVICE in Figure 5, why only mention pre-stimulus alpha power? This is also inconsistent with the abstract saying the "post-stimulus alpha"

Thank you for the commentary, we have corrected the inconsistencies.

With respect to "pre-stimulus" and "post-stimulus" confusion, as can be seen from figure 5. (spectrograms) and figure 8 (CPD decomposition), the alpha power is mostly concentrated before the stimulus and starts to diminish shortly after the stimulus presentation (100-200ms). Since the brain reaction is not instantaneous and for the lack of better word, we've used "pre-stimulus" alpha as it covers the meaning we have intended.

Following your commentary we have changed that to "mostly pre-stimulus alpha" to avoid further confusion.

Line 779: aren't the difference between PRO vs. NOVICE instead of target vs. non-target? Please explain.

Thank you for your comment. We corrected this part. The spectrum was PRO vs. NOVICE.

Line 823: how can the statement be claimed based on Line 825's non-significant results?

Line 823 is about comparison between the PRO and NOVICE groups or intergroup analysis.. In contrast, Line 825 is related to the comparison within the groups or intragroup analysis. In other words, the professional players had shorter reaction times and shorter latencies of the ERPs than the novices. However, within the PRO group, the correlation between the results of RT tests and ERPs parameters was non-significant. A similar situation was observed for the NOVICE group.
We rephrased the corresponding paragraph and separated the part related to the intragroup analysis.

Line 824: spell out the "change" e.g. faster responses
Done

Line 833: could expand on this point, so where does this superiority of PRO in reaction time comes from?

We rephrased this sentence. The new version:
"Thus, the superiority of professional players in reaction time to novices is mostly related to intergroup factors, i.e., the difference between professional and novice players. In other words, the fast reaction can be the necessary cognitive ability for eSport players in CS:GO, which does not have a strong linear link to their immersion and experience in the game. "

Line 848: what were the new findings in addition to previous VS tests?

The main difference between the VS tests in the previous research is that a significant correlation was observed even when visual search tests were conducted separately from EEG experiments. In other words, the participant (PRO and NOVICE) with higher final score during VS test had a statistically significant lower amplitude of N200 peaks in the occipital and parietal regions of their heads.

Line 859: if this statement about experiences is true, the authors should observe a correlation between hours of play and ERP amplitude.

This statement corresponds to the differences between professional players and novices.
The PRO group showed better results in finding the errors during the ST paradigm of the EEG experiments than the NOVICE group. The ERP amplitude of P300 was significantly greater for

the PRO group. However, the VS test results were the same for both groups. In other words, the statement is about intergroup (between the groups) differences in CS:GO experience.
Hours of play, in turn, were calculated within the PRO group and are related to intragroup analysis. This is spelled out in the following subsection “Correspondence of player`s level to the cognitive tests and ERPs” (Section - discussion).
We have updated this part of the text to make it more explicit.

Line 872: consider rephrasing “The absence of a significant correlation between the hours in the game and visual search features, in turn, is expected as the difference between professional and novice players was not observed.“ for clarity.
Done

Line 881: it shouldn’t be titled as the Conclusion since it did not conclude anything about this study.

Thank you for the comment, we renamed this subsection to “Associating eSport with a real sport”

Line 900: please explain the benefits of separating PRO and NOVICE and using EEG to rank the players.

Done. We duplicate the updated text presented at the end of the Conclusion section of the manuscript:
“As for the practical influence of the research, cognitive tests on simple reaction time and choice reaction time can be used to separate professional and novice players by their cognitive skills. This separation can facilitate the selection of the most suitable training plan. The EEG evaluation, in turn, can be used to rank the level of the players more accurately in order to select the most potential players.“
