# Supplementary material for: Biomarkers of professional cybersportsmen: Event related potentials and cognitive tests study
Source: PLoS One. 2023 Aug 1;18(8):e0289293. doi: 10.1371/journal.pone.0289293 (PMC10393144; doi:10.1371/journal.pone.0289293)
Supplement: Attachment — Submitted filename: Response to reviewers.pdf [file pone.0289293.s011.pdf]

Respond letter to the Reviewers' comments

6. Review Comments to the Author

Please use the space provided to explain your answers to the questions above. You may also include additional comments for the author, including concerns about dual publication, research ethics, or publication ethics. (Please upload your review as an attachment if it exceeds 20,000 characters)

Reviewer #3: The authors made a thorough effort to address my previous comments. They included explicit descriptions, tables, supplementary files, and relevant literature, resulting in a significantly enhanced manuscript that exhibits improved clarity in its description, methodology, and presentation of results. Nonetheless, there is room for further improvement in terms of conciseness and overall refinement of the paper. Please see my comments below.

1. Please carefully read through the paper to ensure the sentences are correct and easy to follow.

Thank you for the recommendation. We have reread and updated the manuscript.

2. For example, Line 633 "alpha power before and 200-300ms after the stimulus" is grammatically incorrect. How about changing the (mostly) pre- and post-stimulus alpha-band power to stimulus-locked alpha-band power?

Thank you for the recommendation. Updated.

3. In the Abstract, please rephrase "The comparison of cognitive tests showed statistically significant superiority of professional players in reaction time (faster) and choice reaction time - faster reaction, but similar correctness, while a significant difference in visual search skills was not detected." since the superiority technically could not be statistically significant. I suggest report the results as the metrics you used first, then interpret the results.

Updated.

4. Line 93: Thanks for adding the negative evidence to make the paper more complete. However, connection between GD and other literatures reviewed in the Introduction, especially in the previous and the next paragraph, should be further discussed.

We moved this paragraph to the more appropriate part in the manuscript.

5. Line 197: Need to rephrase the first two sentences. The authors reply to my question about representation; however, it is still not clear in the text. To the best of knowledge, representation is the neural activities that reflect certain cognitive or behavioral processes.

Done.

6. Line 244: Suggest adding temporal domain "of the neural signals" for the first hypothesis.

Thank you for this suggestion. Added.

7. Line 516: I don't think the parenthesis is needed.

Updated.

8. Figure 3: there is an extra blank in 100 000 permutations.

We deleted the extra blank in "100 000" to get "100000".

9. Line 675 & Figure 8: It should be high-er frequency band because 3-30 Hz is not really considered "high-frequency".

Thank you for the suggestion. We have updated the main text and the figure captions.

10. Line 805: What does it mean by "diverse"?

Diverse meant different. The sentence was updated.

11. Line 813: I suggest change the "facts" to findings and "can mean" to "suggest". Please make sure to discuss the findings within the scope of the current.

Updated.

12. Line 850: "Similar" to "Similarly"?

This sentence is correct. We report results similar to other studies.

13. Line 895: "Our suggestion is" to "Our results suggest"

Done.

14. Line 958: This sentence is circular, please rephrase.

Rephrased.

15. Line 966: Please clarify “has another picture”

The clarification of the meaning of “another picture” is written in the next sentence. We decided to merge these two sentences to avoid this unclarity.

16. Line 1055: Please be careful about the distinction between “spectrum” and “spectrogram”. I believe the authors intend to say “spectrogram” as in the Figure 5, please make sure to use correct term throughout the paper. E.g. either take out Figure 5 or the spectrum of the ERPs

Done.

17. Line 1046: The Conclusion (and other parts of the paper) needs to be more precise. Please clearly state the hypothesis in one sentence, and the corresponding results in the next sentence for each point. For example, Hypothesis 4 first few sentences could be shortened to “We only analyze the relations between the cognitive and ERPs since they were recorded separately.” The details should not be included in the Conclusion.

We shortened the conclusion part by rephrasing and omitting some details.
