# Supplementary material for: Biomarkers of professional cybersportsmen: Event related potentials and cognitive tests study
Source: PLoS One. 2023 Aug 1;18(8):e0289293. doi: 10.1371/journal.pone.0289293 (PMC10393144; doi:10.1371/journal.pone.0289293)
Supplement: Attachment — Submitted filename: PLOSONE_review_R2.docx [file pone.0289293.s010.docx]

The authors made a thorough effort to address my previous comments. They included explicit descriptions, tables, supplementary files, and relevant literature, resulting in a significantly enhanced manuscript that exhibits improved clarity in its description, methodology, and presentation of results. Nonetheless, there is room for further improvement in terms of conciseness and overall refinement of the paper. Please see my comments below.

1. Please carefully read through the paper to ensure the sentences are correct and easy to follow. For example, Line 633 “alpha power before and 200-300ms after the stimulus“ is grammatically incorrect.
2. How about changing the (mostly) pre- and post-stimulus alpha-band power to stimulus-locked alpha-band power?
3. In the Abstract, please rephrase “The comparison of cognitive tests showed statistically significant superiority of professional players in reaction time (faster) and choice reaction time - faster reaction, but similar correctness, while a significant difference in visual search skills was not detected.” since the superiority technically could not be statistically significant. I suggest report the results as the metrics you used first, then interpret the results.
4. Line 93: Thanks for adding the negative evidence to make the paper more complete. However, connection between GD and other literatures reviewed in the Introduction, especially in the previous and the next paragraph, should be further discussed.
5. Line 197: Need to rephrase the first two sentences. The authors reply to my question about representation; however, it is still not clear in the text. To the best of knowledge, representation is the neural activities that reflect certain cognitive or behavioral processes.
6. Line 244: Suggest adding temporal domain “of the neural signals” for the first hypothesis.
7. Line 516: I don’t think the parenthesis is needed.
8. Figure 3: there is an extra blank in 100 000 permutations.
9. Line 675 & Figure 8: It should be high-er frequency band because 3-30 Hz is not really considered “high-frequency”.
10. Line 805: What does it mean by “diverse”?
11. Line 813: I suggest change the “facts” to findings and “can mean” to “suggest”. Please make sure to discuss the findings within the scope of the current.
12. Line 850: “Similar” to “Similarly”?
13. Line 895: “Our suggestion is” to “Our results suggest”
14. Line 958: This sentence is circular, please rephrase.
15. Line 966: Please clarify “has another picture”
16. Line 1055: Please be careful about the distinction between “spectrum” and “spectrogram”. I believe the authors intend to say “spectrogram” as in the Figure 5, please make sure to use correct term throughout the paper. E.g. either take out Figure 5 or the spectrum of the ERPs
17. Line 1046: The Conclusion (and other parts of the paper) needs to be more precise. Please clearly state the hypothesis in one sentence, and the corresponding results in the next sentence for each point. For example, Hypothesis 4 first few sentences could be shortened to “We only analyze the relations between the cognitive and ERPs since they were recorded separately.” The details should not be included in the Conclusion.
